# Encoding Light in Electronic Noise

**DOI:** 10.1021/acsnano.6c08996

**Published:** 2026-10-05

**Authors:** Arpan Ghosh, Seema Rani, Anshul Rasyotra, Samriddha Ray, Karthik G. Krishnan, Md Sajjad Alam, Anirban Chowdhury, Kaushik Ghosh, Saptarshi Das

**Affiliations:** † Engineering Science and Mechanics, Penn State University, University Park, Pennsylvania 16802, United States; ‡ 372940 Institute of Nanoscience and Technology, Mohali 140306, India; § Electrical Engineering, Penn State University, University Park, Pennsylvania 16802, United States; ∥ Materials Science and Engineering, Penn State University, University Park, Pennsylvania 16802, United States

**Keywords:** light encoding, RTN, stochasticity, defects, trapping, 2D FETs, scaled oxide

## Abstract

Optical sensing in semiconductors is traditionally governed by photoconductive, photovoltaic, and photogating effects, in which optical inputs are encoded as continuous variations in average current. Here, we demonstrate a fundamentally distinct paradigm in which light is encoded in stochastic transport dynamics rather than steady-state conductance. Using a monolayer MoS_2_ field-effect transistor with an aggressively scaled gate stack (∼1.3 nm equivalent oxide thickness), we access a regime where interfacial defect states dominate transport and form gate-tunable potential wells. At low temperature, carrier emission from these states is suppressed, stabilizing trapped configurations in the dark, whereas optical excitation selectively activates emission, giving rise to large-amplitude (“giant”) random telegraph noise. This enables independent control of capture and emission processes through electrostatic gating and illumination, respectively, creating a direct mapping between optical input and stochastic observables such as switching rates, dwell times, and state occupancies. Interestingly, optical detection is achieved through the statistics of rare emission events, enabling sensitivity to ultralow illumination levels (∼50 fW). Spectral dependence further reveals photon energy–dependent emission kinetics, confirming the role of photon-assisted detrapping. These results show a light-induced nonequilibrium transport regime in which defect-mediated fluctuations, conventionally treated as noise, act as a functional signal and provide a stochastic sensing framework for ultralow-light detection in nanoscale electronic systems.

## Graphical abstract

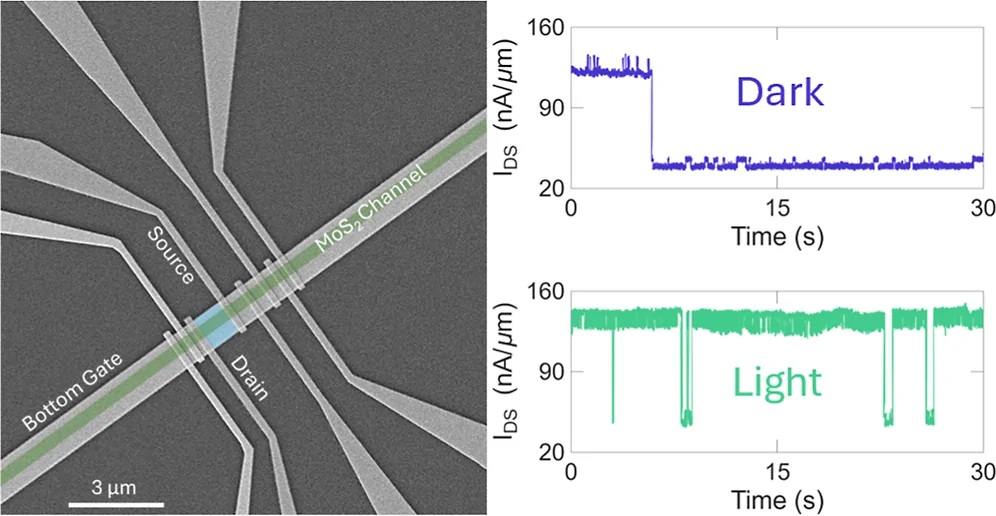

## Introduction

Optical sensing in semiconductors is conventionally based on measuring changes in the average current arising from photogenerated carriers. Within this framework, stochastic fluctuations are treated as noise that limit sensitivity and precision. However, as device dimensions approach the nanoscale, charge transport becomes inherently stochastic, often dominated by discrete trapping and detrapping events at localized defect sites. This raises a fundamental question: Can noise itself serve as a functional signal for sensing?

In low-dimensional systems, particularly two-dimensional (2D) semiconductors, reduced screening and strong electrostatic coupling to the surrounding dielectric environment amplify the influence of individual defects on transport.^1,2^ These systems frequently exhibit random telegraph noise (RTN), arising from stochastic capture and emission of carriers at interfacial trap states.^3–5^ While RTN is typically viewed as a source of variability and instability, it encodes detailed information about microscopic charge dynamics and reflects a nonequilibrium transport regime governed by transition kinetics rather than steady-state carrier populations.^6–8^ Despite the prevalence of such defect-mediated fluctuations, their role under optical excitation has remained largely unexplored as a sensing modality.

Conventional photodetection mechanisms, including photoconductive, photovoltaic, and photogating effects, encode optical information in quasi-static or continuous changes in conductance.^9–12^ Even in transistor architectures where photogating can amplify responses through trapped charges, detection ultimately relies on shifts in the mean current or threshold voltage.^13–15^ In contrast, stochastic transport offers a fundamentally different route to sensing, in which information is encoded in temporal statistics such as switching rates and dwell times.^16^ Such an approach is particularly attractive in regimes of ultralow illumination, where average signals become vanishingly small but rare stochastic events remain observable.

Here, we demonstrate a stochastic sensing paradigm in which optical signals are encoded in defect-mediated switching dynamics rather than mean conductance. Using a monolayer MoS_2_ field-effect transistor (FET) with an aggressively scaled gate stack (equivalent oxide thickness, EOT ∼ 1 nm), we access a regime where interfacial defect states form gate-tunable potential wells that localize carriers at low temperature. In the dark, carrier emission from these states is strongly suppressed, stabilizing trapped configurations. Under illumination, photon-assisted emission activates transitions between trapped and untrapped states, giving rise to large-amplitude “Giant” RTN. This enables independent control of capture and emission processes through electrostatic gating and optical excitation, creating a direct mapping between optical input and stochastic observables. Crucially, detection is achieved through the statistics of rare emission events, enabling sensitivity to ultralow light levels (∼50 fW). The emission kinetics further exhibit a clear dependence on photon energy, enabling spectrally sensitive response within the same stochastic framework. By harnessing defect-mediated fluctuations as a functional signal, this work establishes a light-induced nonequilibrium transport regime and produces a new framework for probabilistic sensing in ultrascaled electronic systems.

## Results and Discussion

### Observation of “Giant” Stochastic Switching in Scaled Gate Stack under Illumination

We begin by describing the device architecture and measurement framework used to probe stochastic transport in MoS_2_ FET under optical excitation. Figure 1a and Supporting Information Figure S1, respectively, show the schematic and scanning electron micrograph (SEM) of a monolayer MoS_2_ FET integrated with an aggressively scaled gate stack (Ti/Pt/5 nm HfO_2_), corresponding to an equivalent oxide thickness (EOT) of ∼1.3 nm, and Ni/Au source/drain contacts. Supporting Information Figure S2 shows the Raman and photoluminescence (PL) spectra for the MoS_2_ channel grown via metal organic chemical vapor deposition (MOCVD), confirming its monolayer thickness.^17,18^ Supporting Information Figure S3 presents the device schematic and capacitance–area scaling of metal–insulator–metal structures used to extract the EOT of the gate stack.^19^ The combination of reduced channel dimensionality and an ultrathin gate dielectric enables strong electrostatic coupling between the channel and the dielectric interface, rendering transport highly sensitive to interfacial defect states. Details of material synthesis,^20–22^ device fabrication,^23,24^ and electrical characterization^25^ can be found in our earlier work and in the Methods Section.

**Figure 1:**
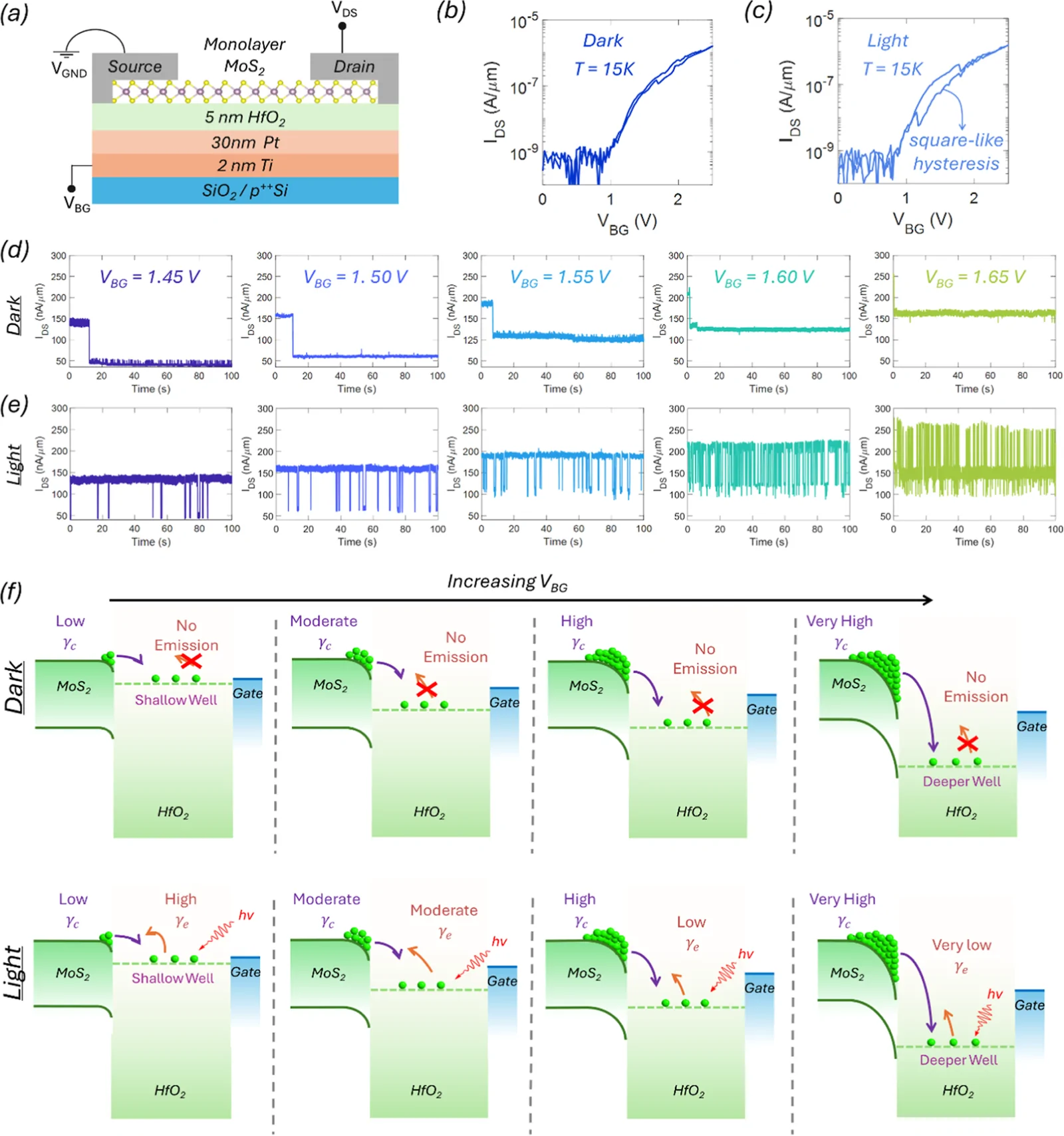
Stochastic photo sensing using MoS_2_ field effect transistor (FET) with aggressively scaled gate stack. (a) Schematic of a back-gated monolayer MoS_2_ FET with 5 nm HfO_2_ as back-gate dielectric. Back-gate transfer characteristics, i.e., source to drain current (*I*_DS_) measured by sweeping the back-gate voltage (*V*_BG_) at a constant source-to-drain voltage, *V*_DS_ = 1 V in (b) dark and (c) under illumination at 15 K. Time-resolved *I*_DS_ traces recorded at different *V*_BG_ in (d) dark and (e) under illumination. In dark, a single transition to a lower-current state is observed, whereas illumination drives repeated switching between well-defined upper and lower current states, resulting in photoactivated giant random telegraph noise (RTN). (f) Schematic illustrating the physical mechanism of stochastic photo sensing. Increasing *V*_BG_ increases the carrier density in the MoS_2_ channel and enhances capture rate (γ_c_) into interfacial trap states, while simultaneously deepening the trap potential. In the dark, emission is suppressed, leading to stable trapped configurations. Under illumination, optical excitation increases emission rate (γ_e_) from trap states, enabling dynamic switching between trapped and untrapped states. The interplay between electrostatically controlled γ_c_ and illumination controlled γ_e_ governs the observed stochastic dynamics.

We first examine the quasi-static transport characteristics under dark and illuminated conditions at 15 K using low-intensity illumination from a light-emitting diode (LED), representative of realistic, low-light conditions rather than high-intensity laser excitation. Electrical measurements are performed in a back-gated configuration, where the gate voltage *V*_BG_ modulates the carrier density (*n*_s_) in the MoS_2_ channel, and the drain current *I*_DS_ is measured at a fixed drain bias *V*_DS_ = 1 V. The resulting *I*_DS_ – *V*_BG_ characteristics define the transfer curves of the device. Figure 1b,c shows representative transfer curves in the dark and under illumination at 15 K, respectively, while Supporting Information Figure S4 compiles multiple consecutive sweeps from the same device to confirm reproducibility. In the dark, the transfer curves are relatively smooth and largely reproducible across gate voltage sweeps, with only occasional weak fluctuations reflecting intrinsic RTN in localized regions and minimal hysteresis. Under illumination, however, the transfer characteristics develop pronounced irregularities and square-like hysteresis, with abrupt transitions between distinct current levels that vary between successive sweeps, particularly in the subthreshold regime. This behavior reflects enhanced stochastic switching between discrete conductance states occurring during the measurement and provides a clear signature of light-activated RTN dynamics. In contrast, at room temperature, the transfer characteristics are smooth and nearly identical under dark and illuminated conditions, as shown in Supporting Information Figure S5, with both exhibiting negligible hysteresis. This behavior is consistent with prior reports in MoS_2_ FETs, where low-intensity illumination using an LED produces negligible photoconductive response.^26–29^ While photogating effects are commonly observed in such devices, they typically require operation in the deep off-state and arise from quasi-static charge trapping that modulates the channel electrostatically, rather than manifesting in stochastic switching dynamics. The absence of pronounced hysteresis reflects the collective effect of a broad ensemble of trap states spanning a wide range of time and energy scales, leading to temporal and energetic averaging of trapping–detrapping processes.^30,31^ Together, these results highlight a transition from quasi-static, ensemble-averaged transport at room temperature to light-activated stochastic transport at low temperature, proving that the observed dynamics originate from photon-assisted emission processes rather than conventional photoconductive effects.

To directly probe these dynamics, we monitor the temporal evolution of the drain current *I*_DS_(*t*) at fixed bias conditions at 15 K. Figure 1d,e shows representative time traces as a function of *V*_BG_, in dark and under illumination (*P*_IN_ = 17 pW) from a white LED. In the absence of illumination, the current remains largely stable over time, with a single transition into a lower current state. These events correspond to carrier capture into an ensemble of interfacial defect states, after which the system predominantly remains in a trapped configuration, indicating that emission is suppressed within the experimental time scale. Temperature-dependent photoluminescence (PL) measurement supports the presence of defect states which mediate trapping. As shown in Supporting Information Figure S6, in addition to the dominant MoS_2_ emission near ∼1.9 eV, a distinct lower-energy emission feature around ∼1.78 eV, corresponding to X^L^ excitons, becomes increasingly pronounced as the temperature is reduced. This feature is strongly suppressed with increasing temperature and becomes indistinguishable at 300 K. Such a thermally quenched, lower-energy emission feature is consistent with radiative recombination involving localized defect- or trap-associated states.^32–34^ In contrast, the higher-energy spectral contribution near ∼2.05 eV becomes more prominent with increasing temperature, further demonstrating the strong temperature dependence of the underlying recombination pathways. Under illumination, however, repeated switching between two well-defined current levels emerges, giving rise to “Giant” RTN. The current fluctuates between a higher level, *I*_TU_ (trap unoccupied), and a lower level, *I*_TO_ (trap occupied), with a characteristic amplitude Δ*I*_DS_ = *I*_TU_ – *I*_TO_. Notably, the RTN amplitude (∼100 nA) is unusually large compared to conventional reports in nanoscale transistors.^35^

The gate dependence of these dynamics reflects the interplay of electrostatic control and optical activation in the trapping process. As *V*_BG_ increases, transitions into the trapped state occur progressively earlier in the dark, indicating a reduction in the capture time, τ_c_. This behavior can be understood by considering the capture rate, γ_c_ = 1/τ_c_ into interfacial defect states, given by, γ_c_ = σ*v*_th_ *n*_s_, where σ is the capture cross-section, *v*_th_ is the carrier thermal velocity, and *n*_s_ is the channel carrier density.^36^ In the subthreshold regime, the carrier density depends exponentially on gate voltage following eq 1

1 $$n_{s}\propto \exp\left(\frac{V_{BG}-V_{TH}}{SS}\right),,SS=m\frac{k_{B}T}{q}$$

where SS is the subthreshold swing, *V*_TH_ is the threshold voltage, *m* is the band movement factor, *k*_B_ is the Boltzmann constant, *T* is the temperature, and *q* is the electronic charge. This should lead to an exponential dependence of τ_c_ on *V*_BG_. However, experimentally, we observe an approximately linear dependence of the capture dynamics on *V*_BG_ (Supporting Information Figure S7), suggesting that additional mechanisms, such as trap energetics, spatial distribution, or field-dependent capture cross sections, modulate the effective capture process beyond the ideal subthreshold model. Nevertheless, increasing *V*_BG_ consistently enhances the capture rate and reduces τ_c_, in agreement with the earlier onset of trapping observed in the time-domain measurements.

The persistence of bidirectional switching under illumination indicates that optical excitation activates carrier emission, enabling dynamic transitions between the trapped and untrapped states. As illustrated schematically in Figure 1f, the applied gate bias defines a potential landscape in which interfacial defects form trap wells that deepen with increasing *V*_BG_. While this enhances carrier capture, low temperature suppresses thermal emission, stabilizing the trapped state in the dark. The emission process under illumination can be described by a photon-assisted rate, γ_e_, given by eq 2

2 $$\gamma_{e}\propto \sigma_{opt}\Phi_{opt}\exp\left(\frac{E_{T}\left(V_{BG}\right)}{k_{B}T_{eff}}\right)$$

where σ_opt_ is the optical cross-section, Φ_opt_ is the photon flux, and *E*_T_(*V*_BG_) is the gate-dependent trap depth. As *V*_BG_ increases, the trap depth increases, reducing the emission rate, γ_e_, and leading to a longer emission time τ_e_ = 1/γ_e_. Together with γ_c_, this defines a unified two-state system in which the steady-state occupation probabilities for trap occupied (*p*_TO_) and trap unoccupied (*p*_TU_) states are given by eq 3.

3 $$p_{TO}=\frac{\gamma_{c}}{\gamma_{c}+\gamma_{e}},,p_{TU}=\frac{\gamma_{e}}{\gamma_{c}+\gamma_{e}}$$

Correspondingly, the expected current level is given by eq 4.

4 $$\left\langle I_{DS}\right\rangle =p_{TU}I_{TU}+p_{TO}I_{TO}$$

At low *V*_BG_, the emission rate dominates (γ_e_ > γ_c_), resulting in a population inversion with the untrapped state (*I*_TU_) more frequently occupied, i.e., ⟨*I*_DS_⟩ ∼ *I*_TU_. As *V*_BG_ increases, the combined effect of enhanced capture and suppressed emission leads to γ_c_ > γ_e_, driving a reversal in occupancy. Consequently, the system spends progressively more time in the trapped state, i.e., ⟨*I*_DS_⟩ ∼ *I*_TO_, at higher gate bias.

### Optical Control of Stochastic Dynamics

To quantify the role of optical excitation in modulating stochastic transport, we examine the temporal current response *I*_DS_(*t*) at a fixed gate bias (*V*_BG_ = 1.6 V) while systematically varying the incident optical power *P*_IN_, which accordingly changes the photon flux (Φ_opt_). Representative time traces are shown in Figure 2a with increasing *P*_IN_. At low illumination intensity, switching events are sparse, and the system remains predominantly in one state, consistent with weak photon-assisted emission. As *P*_IN_ and consequently Φ_opt_ increases, the switching rate rises, leading to more frequent transitions between the trapped (*I*_TO_) and untrapped (*I*_TU_) states, and eventually to dense stochastic fluctuations at high optical power. Zoomed-in traces over shorter time windows further highlight this evolution, showing that at higher *P*_IN_, the system exits the trapped state more readily, reflecting enhanced photon-assisted emission and reduced dwell times in the trapped configuration.

**Figure 2:**
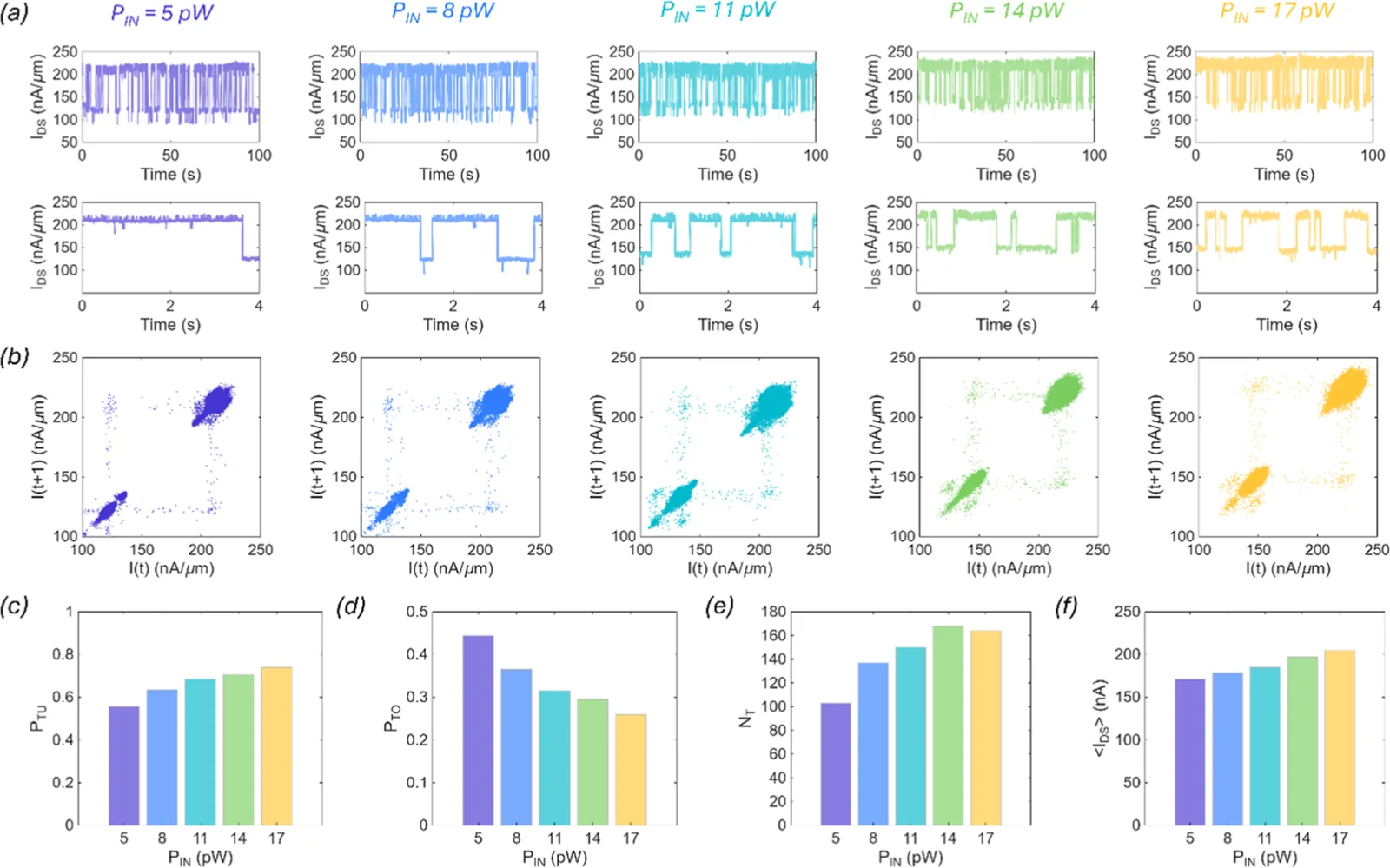
Intensity dependence of stochastic photosensing. (a) Time-resolved *I*_DS_ traces recorded under progressively increasing illumination power (*P*_IN_) at a fixed *V*_BG_ = 1.6 V. Both longer dwell traces (top row) and shorter dwell windows (lower row) are shown to highlight the evolution of the switching dynamics. (b) Time-lag plots (TLPs), i.e., *I*(*t* + Δ*t*) versus *I*(*t*) corresponding to the long-duration traces in (a), show clear bimodal clustering, a characteristic of two-state fluctuations. At lower *P*_IN_, the off-diagonal clusters are sparsely populated, indicating fewer state transitions. With increasing *P*_IN_, off-diagonal population becomes denser, confirming a higher transition frequency. The occupancy probabilities for (c) trap-occupied (*p*_TO_) and (d) trap-unoccupied (*p*_TU_) states for different *P*_IN_. (e) Total number of transitions, *N*_T_ versus *P*_IN_, shows a monotonic increase consistent with an increased frequency of combined carrier capture and emission events under stronger optical excitation. (f) Expected drain current ⟨*I*_DS_⟩ as a function of *P*_IN_. The rise in ⟨*I*_DS_⟩ originates from the longer dwell time of the carrier in the trap-unoccupied (high-current) state at higher illumination intensities.

The corresponding time-lag plots shown in Figure 2b, i.e., *I*(*t* + Δ*t*) versus *I*(*t*), where Δ*t* is the sampling interval, provide a compact visualization of the transition dynamics. At low *P*_IN_, the distribution is concentrated into two clusters with minimal off-diagonal population, indicating infrequent transitions. With increasing *P*_in_, the density of off-diagonal points increases, directly reflecting enhanced transition activity. This evolution captures the transition from a quasi-static regime to a dynamically switching stochastic system driven by optical excitation. We quantify these dynamics through statistical observables extracted from the time traces. The occupancy probabilities *p*_TO_ and *p*_TU_ (Figure 2c,d) directly reflect the relative balance between capture and emission rates. As *P*_IN_ increases, the emission rate γ_e_ increases, shifting the system toward the untrapped state (*p*_TU_↑, *p*_TO_↓). The total number of transitions *N*_T_ (Figure 2e) correspondingly increases, consistent with the expected dependence on the combined transition rates. The expected current, ⟨*I*_DS_⟩, as shown in Figure 2f, follows the same trend, increasing with *P*_IN_ due to the higher probability of the untrapped (high-current) state. We perform the same analysis at a lower gate bias (*V*_BG_ = 1.5 V) in Supporting Information Figure S8 and observe qualitatively similar trends. However, the reduced trap depth facilitates carrier emission, resulting in a higher occupancy of the untrapped state across all *P*_IN_. This is accompanied by a lower number of transitions due to the reduced capture rate at lower carrier density, consistent with the electrostatic control of γ_c_. To showcase the repeatability of the optically induced stochastic dynamics, we measured 10 independent devices under different optical illumination. As shown in Supporting Information Figure S9, all ten devices exhibit light-activated random telegraph noise (RTN), and the emission rate consistently increases with increasing illumination intensity, demonstrating that the key stochastic photosensing behavior is reproducible and not limited to a single representative device. In addition to the device repeatability, we have evaluated the temporal stability of the phenomenon. Supporting Information Figure S10 presents repeated measurements acquired over time, showing that the device consistently exhibits RTN under optical illumination. These results confirm the stability and repeatability of the proposed stochastic sensing mechanism over the duration of our measurements.

### Limit of Detection and Emission Dynamics at Low Optical Power

To evaluate the sensitivity limit of this stochastic sensing paradigm, we investigate emission dynamics at low optical power by reducing *P*_IN_ while operating at a lower gate bias. A reduced *V*_BG_ is selected to balance competing requirements: sufficiently shallow trap potentials to enable photon-assisted emission at low *P*_IN_, while maintaining suppressed thermal detrapping. At excessively low *V*_BG_, thermal emission dominates, whereas at higher *V*_BG_, increased trap depth leads to prohibitively long waiting times for emission events under weak illumination.

Under these optimized conditions, we resolve clear photon-assisted detrapping events down to *P*_IN_ ∼ 50 fW. The time traces in Figure 3a show a systematic decrease in τ_e_ with increasing *P*_IN_, manifested as progressively shorter dwell times in the trapped state. Within this framework, the emission rate is governed by photon-assisted processes, following eq 5.

5 $$\tau_{e}=\frac{1}{\gamma_{e}}\propto \frac{1}{\Phi_{opt}\left(P_{IN}\right)}$$

**Figure 3:**
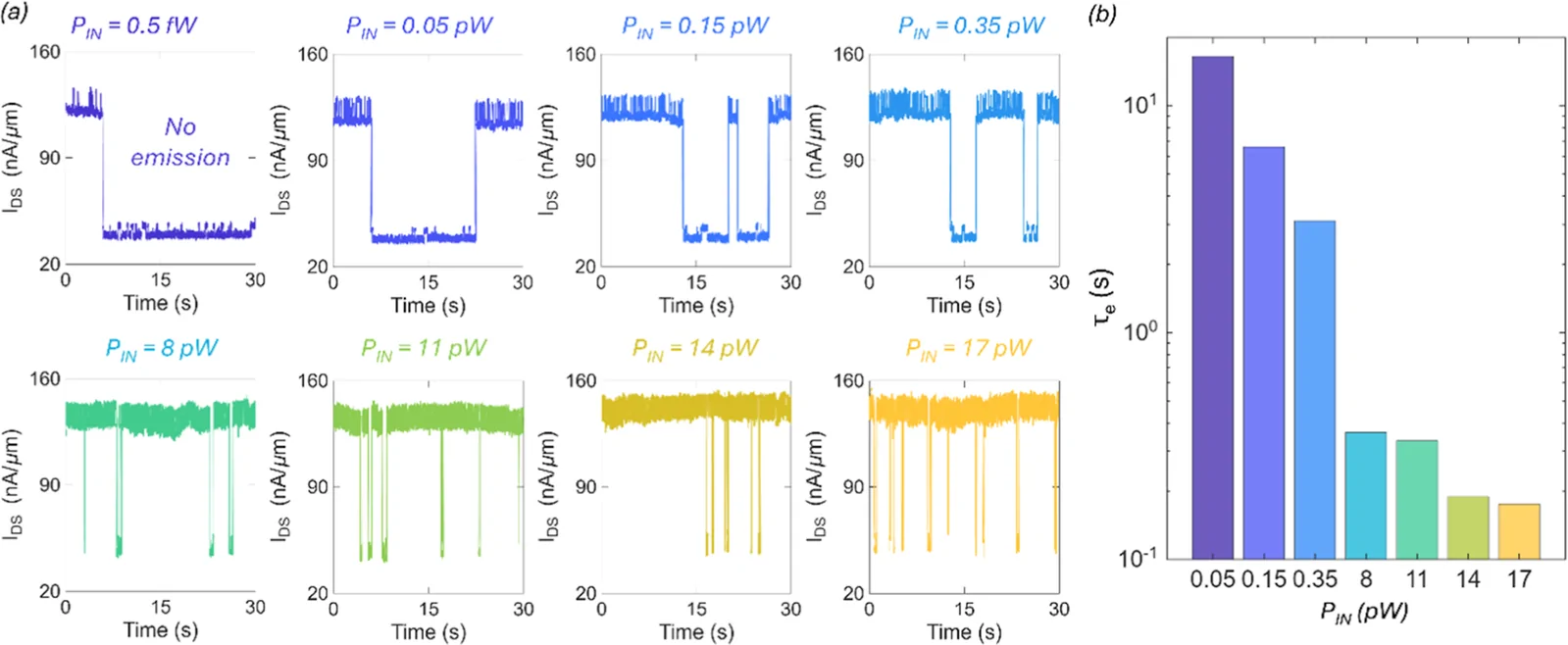
Limit of detection in stochastic photo sensing. (a) Time-resolved *I*_DS_ traces measured at a fixed back-gate bias (*V*_BG_ = 1.45 V) under increasing optical power density, *P*_IN_. At the lowest illumination level (*P*_IN_ = 0.0001 mW cm^–2^) no emission event is observed within the measurement window. As *P*_IN_ increases, emission events become faster and more frequent, reflecting enhanced photon-assisted detrapping. (b) Dwell time in trapped state (τ_e_) as a function of *P*_IN_. The monotonic decrease in τ_e_ with increasing *P*_IN_ indicates that stronger optical excitation enhances the carrier emission rate from the trap states, thereby reducing the residence time in the captured state.

Thus, reducing optical power directly suppresses the emission rate, leading to longer waiting times between detrapping events and hence higher τ_e_. In the low-intensity regime, emission events become rare and can be described as a stochastic Poisson process. The probability of observing at least one emission event within an observation window *T*_obs_ is given by eq 6

6 $$P\left(\geq 1\;event\right)=1-\exp\left(-{\gamma_{e}T}_{obs}\right)$$

which defines the detection limit in this framework. Even when the average current remains effectively unchanged, the presence of rare emission events enables reliable detection through temporal statistics.

We extract τ_e_ as a function of *P*_IN_ from the dwell-time distributions (Figure 3b) and observe a monotonic increase in capture time with decreasing illumination intensity, consistent with the reduction in γ_e_. Notably, emission events remain resolvable within the experimental time window even at *P*_IN_ ∼ 50 fW, obtaining the lower bound of detectable optical input. Unlike conventional photodetection, which relies on changes in mean current, this approach leverages the statistics of infrequent transitions, enabling sensing in regimes where average signals are effectively unchanged. We have also benchmarked our mechanism with other material system, which has been summarized in Supporting Information Table S1.

### Spectral Dependence of Stochastic Switching Dynamics

To probe the energy dependence of photon-assisted emission, we investigated the stochastic dynamics under illumination with red, green, and blue LEDs. The incident optical power, *P*_IN_ = 7.5 pW, is kept constant for each wavelength to isolate the effect of photon energy. By tuning the gate bias *V*_BG_, we modulate the depth of the interfacial trap potential *E*_T_(*V*_BG_), enabling direct control of the emission barrier.

Figure 4a shows representative time traces of *I*_DS_(*t*) under different wavelengths and gate biases. For a fixed *V*_BG_, higher-energy photons (blue) produce significantly more frequent switching compared to lower-energy photons (red), indicating enhanced photon-assisted emission. This behavior reflects a reduction in the effective emission barrier with increasing photon energy, resulting in higher emission rates and shorter dwell times in the trapped state. Within this framework, the emission rate can be described as

7 $$\gamma_{e}\propto \Phi \left(P_{IN}\right)\exp\left(-\frac{E_{T}\left(V_{BG}\right)-h\nu }{k_{B}T_{eff}}\right)$$

where *h*ν is the photon energy and *k*_B_ *T*_eff_ captures the nonequilibrium energy distribution of carriers under optical excitation. The spectral dependence is further reflected in the state occupancy probabilities (Figure 4b,c), where increasing photon energy shifts the system toward the untrapped (high-current) state, resulting in higher *p*_TU_ and reduced *p*_TO_. In contrast, increasing *V*_BG_ deepens the trap potential, suppressing emission, and shifting the system toward the trapped configuration. These competing effects of photon energy and electrostatic control govern the observed stochastic dynamics.

**Figure 4:**
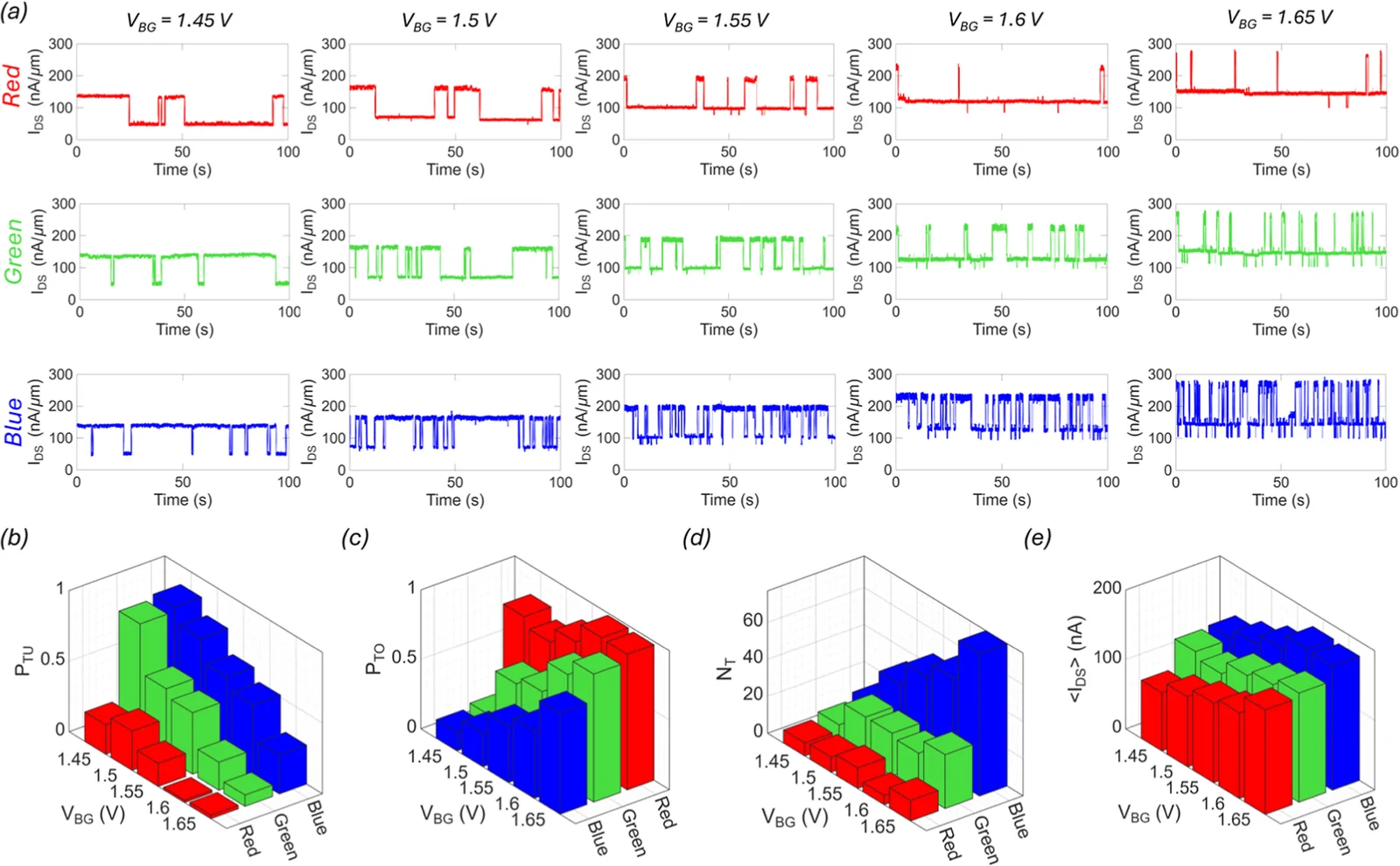
Spectral response of stochastic photo sensing. (a) Time-resolved *I*_DS_ traces measured under illumination with red, green, and blue light at different gate biases (*V*_BG_) and a fixed drain bias (*V*_DS_ = 1 V). For a given *V*_BG_, higher-energy photons (blue) induce more frequent switching events than lower-energy photons (red), consistent with enhanced photon-assisted emission. Corresponding state occupancy probabilities for (b) trap-unoccupied (*p*_TU_) and (c) trap-occupied (*p*_TO_) states. (d) Total number of state transitions (*N*_T_) and (e) time-averaged current, ⟨*I*_DS_⟩, extracted over a 100 s measurement window for different wavelengths and gate biases.

The corresponding time-lag plots (Supporting Information Figure S11) provide a compact visualization of the switching dynamics and directly reflect the transition activity: higher photon energy increases the density of off-diagonal points, indicating enhanced switching, whereas increasing *V*_BG_ reduces this density, consistent with suppressed emission. These trends are quantitatively captured by the total number of transitions, *N*_T_, shown in Figure 4d, which increases with photon energy and *V*_BG_. Finally, Figure 4e shows ⟨*I*_DS_⟩, which increases with photon energy due to the higher probability of the untrapped (high-current) state for any given *V*_BG_. Together, these results show that stochastic dynamics arise from the interplay between photon energy and electrostatically controlled trap depth, enabling a spectrally sensitive response.

### Interplay of Thermal Activation and Electrostatic Control

To further elucidate the underlying dynamics, we examine the temperature dependence of the stochastic switching behavior (Supporting Information Figure S12). At low temperatures (15 K), pronounced two-level switching is observed across a broad range of gate biases, consistent with suppressed thermal emission and dominance of photon-assisted detrapping. As the temperature increases, the switching activity becomes progressively more intermittent and eventually diminishes, particularly at higher *V*_BG_, where the system remains predominantly in the trapped state with only occasional emission events. At lower *V*_BG_, elevated temperature enhances detrapping, leading to reduced dwell times in the trapped state and, in some cases, partial smearing of the two-level fluctuations due to increased background conductance.

Within the framework, this behavior arises from the temperature dependence of the emission rate described using eq 7, where the first term captures photon-assisted emission and the second term represents thermally activated detrapping. At low temperatures, thermal emission is negligible, and γ_e_ is dominated by optical excitation, resulting in well-defined stochastic switching. As temperature increases, the thermal contribution becomes significant, effectively increasing the baseline emission rate and reducing the contrast between optically driven and thermally activated processes. At room temperature, this leads to the disappearance of discrete switching, with no resolvable RTN due to rapid, thermally averaged trapping–detrapping dynamics (Supporting Information Figure S13). At high *V*_BG_, where *E*_T_ is large, both thermal and optical emission are suppressed, leading to reduced switching activity even at elevated temperatures. Overall, these results confirm that stochastic switching is governed by a competition between electrostatically controlled trap depth and temperature- and photon-dependent emission pathways, with temperature providing an additional tuning knob that modulates the relative contributions of thermal and optical detrapping.

### Quantifying Defect Ensemble Size from Stochastic Switching

This framework also enables an estimate of the number of participating trap states directly from the measured current fluctuations. Note that the device operates in the subthreshold regime, where the drain current depends exponentially on gate voltage following eq 1. Therefore, the observed current modulation can be directly mapped to an effective threshold voltage shift Δ*V*_TH_, following eq 8

8 $$\Delta V_{TH}=SS\times \ln\left(\frac{I_{TU}}{I_{TO}}\right)$$

This shift corresponds to a change in charge Δ*Q* = *C*_OX_Δ*V*_TH_, where *C*_OX_ is the gate capacitance per unit area, and the effective number of charges involved in a switching event is given by *N* = Δ*Q*/*q*. Using the experimentally observed *I*_TU_ and *I*_TO_, we extract Δ*V*_TH_ ∼ 150 mV and obtain *N* ∼ 12,500, indicating that the stochastic switching arises from a localized ensemble of strongly coupled trap states rather than a single defect. Notably, this estimate captures the order of magnitude of the participating defect population without explicitly accounting for its detailed dependence on *V*_BG_.

## Conclusion

In summary, we demonstrate a fundamentally different paradigm for photodetection in which optical information is encoded in stochastic current fluctuations rather than changes in mean conductance. By leveraging aggressively scaled electrostatics in 2D FETs, we access a regime where interfacial defect states form gate-tunable potential landscapes that govern carrier trapping and emission. Optical excitation selectively activates emission, while electrostatic control modulates capture and trap depth, enabling independent tuning of the underlying transition kinetics. This framework creates a direct mapping between optical inputs and stochastic observables, including state occupancy, transition activity, and dwell-time statistics. The observed dependence on optical power, combined with sensitivity to photon energy, highlights the versatility of this approach. Notably, detection is achieved down to optical intensities of ∼50 fW through the statistics of rare events, enabling operation in regimes where conventional photodetection fails due to negligible changes in average current. More broadly, these results reveal that defect-mediated fluctuations, typically regarded as noise, can serve as a functional signal in ultrascaled electronic systems. By shifting the sensing modality from deterministic averages to stochastic dynamics, this work opens new opportunities for probabilistic optical sensing and suggests a general framework for harnessing noise in electronic and optoelectronic devices.

## Methods

### Synthesis of MoS_2_

Monolayer MoS_2_ films were grown by metal–organic chemical vapor deposition (MOCVD) on prescored, double-side-polished 2-in. *c*-plane sapphire substrates. The deposition was carried out in a cold-wall horizontal reactor equipped with an inductively heated graphite susceptor and continuous wafer rotation to promote uniform thickness across the substrate. Molybdenum hexacarbonyl (Mo­(CO)_6_) and hydrogen sulfide (H_2_S) were used as the molybdenum and sulfur precursors, respectively. Mo­(CO)_6_ was supplied from a stainless-steel bubbler maintained at 10 °C and 950 Torr, with a flow rate of 0.036 sccm, while H_2_S was introduced at 400 sccm. Prior to growth, the sapphire substrates were annealed in H_2_ at 1000 °C for 10 min. The MoS_2_ deposition was then performed at 1000 °C and 50 Torr for 18 min under a hydrogen atmosphere. Following growth, the samples were cooled to 300 °C in an H_2_S atmosphere to suppress film decomposition.

### Device Fabrication

Locally back-gated island architectures were fabricated on commercially available 285 nm SiO_2_/p^++^-Si substrates. A bilayer electron-beam resist stack consisting of PMMA EL6 as the first layer and PMMA A3 as the second layer was first spin-coated at 4000 rpm for 45 s, followed by sequential soft-bake steps at 150 and 180 °C for 90 s each, respectively. The geometries were then defined by electron-beam lithography (EBL), and the exposed resist was developed in a 1:1 MIBK/IPA solution for 60 s, followed by an IPA rinse for 45 s. Back-gate electrodes were formed by depositing 2 nm Ti and 30 nm Pt using electron-beam evaporation in a Temescal FC-2000 system. Excess metal was removed by lift-off in acetone, followed by IPA rinsing. Subsequently, a 5 nm HfO_2_ dielectric layer was deposited by atomic layer deposition at 200 °C to serve as the back-gate insulator. Openings to access the individual gate electrodes were then created by reactive-ion etching using a PlasmaTherm Versalock 700 system. In the following step, contact pad regions were patterned through a second EBL process. The ZEP-520A resist was spin-coated at 2500 rpm for 45 s and baked at 180 °C for 3 min. After electron-beam exposure, the resist was developed in *n*-amyl acetate. 5 nm HfO_2_ dielectric within the pad-opening regions was selectively etched using BCl_3_ plasma at 5 °C for 15 s. Finally, the residual resist was stripped using PRS-3000, and the substrate was cleaned with IPA, completing the fabrication of the patterned local back-gate structures. To fabricate the MoS_2_ FETs, the MOCVD-grown monolayer MoS_2_ was transferred from the sapphire growth substrate onto the SiO_2_/p^++^-Si substrate containing local back-gate islands using a PMMA-assisted wet-transfer method. The process started with spin-coating the PMMA support layer onto the MoS_2_/sapphire sample, which was then stored in a nitrogen cabinet overnight to ensure proper adhesion and drying. The edges of the PMMA-coated film were gently scored using a razor blade before immersing the sample in deionized water maintained at 90 °C. The water penetrated the MoS_2_/sapphire interface through capillary action, enabling the PMMA/MoS_2_ stack to delaminate from the growth substrate. After separation, the floating film was sequentially cleaned in multiple deionized water baths. The cleaned PMMA/MoS_2_ layer was then carefully transferred onto the target SiO_2_/p^++^-Si substrate with predefined local back-gate islands. To complete the transfer, the sample was baked sequentially at 50 and 70 °C for 20 min each, which helped eliminate trapped moisture and residual transfer-related contaminants, thereby promoting a clean and uniform interface. The PMMA was later removed in acetone, and the sample is rinsed in IPA. The active channel regions of MoS_2_ transistors were defined by electron-beam lithography following spin-coating of PMMA resist and a soft bake at 180 °C for 90 s. After exposure, the resist was developed in a 1:1 MIBK/IPA solution, and the exposed MoS_2_ regions were selectively removed by SF_6_ plasma etching at 5 °C for 13 s. The samples were subsequently cleaned with acetone and IPA to remove the residual resist, thereby isolating the transistor channels. Following the channel definition, aligned source and drain electrodes were fabricated. To improve resist wetting and coating uniformity, the substrates were first treated in Surpass 4 K for 60 s, rinsed with deionized water, and baked at 100 °C for 60 s. PMMA resist was then spin-coated at 5000 rpm for 45 s and baked at 180 °C for 3 min. Electron-beam lithography was carried out at an acceleration voltage of 100 keV, followed by development in a 1:1 MIBK/IPA solution at room temperature. After contact patterning, 15 nm Ni/25 nm Au were deposited by electron-beam evaporation. For contacts, lift-off was performed in acetone followed by IPA rinsing, completing the formation of self-aligned source–drain contacts.

### Scanning Electron Microscopy

Scanning electron microscopy (SEM) of the 2D MoS_2_ transistors used in this study was taken using a Zeiss Gemini 500 field-emission scanning electron microscopy (FESEM) system at an accelerating voltage of 5 kV.

### Raman and Photoluminescence Spectroscopy

Raman and photoluminescence (PL) spectra measurement was acquired on a Horiba LabRAM HR Evolution confocal Raman microscope, using a 532 nm laser set to 34 mW, filtered at 1%. The setup included a 100× objective with a numerical aperture of 0.9 and gratings with spacings of 1800 gr mm^–1^ and 300 gr mm^–1^ for Raman and PL respectively.

### Electrical Characterization

Electrical characterization of the MoS_2_ FET devices was performed using a Lake Shore CRX-VF probe station under high-vacuum conditions and low temperature with a Keysight B1500A parameter analyzer. For high-speed measurements, a Keysight PZ2100A high-density precision source measure unit (SMU) mainframe with PZ2121A high-speed SMUs was used. A continuous-wave white light LED source was used for all experiments involving light illumination unless otherwise stated.

## Supplementary Material



## Data Availability

Data sets generated during and/or analyzed during the current study and the codes used for plotting the data are available from the corresponding authors upon reasonable request.

## Notes

SEM of back-gated MoS_2_ FET, Raman and photoluminescence characterization of MoS_2_, Metal–insulator–metal structure along with dielectric characterization, transfer characteristics of MoS_2_ FET under dark and illumination at 15 K, transfer characteristics of MoS_2_ FET under dark and illumination at room temperature, temperature-dependent PL of MoS_2_/HfO_2_ interface, back gate bias dependent time of capture, intensity dependence of stochastic photo sensing, multiple MoS_2_ FETs showcasing the stochastic light-induced RTNs, temporal stability of light-induced RTN, time lag plots for visualizing the wavelength and gate bias dependence of stochastic dynamics in MoS_2_ FET, temperature dependence of stochastic switching dynamics, stochastic switching dynamics at room temperature, and benchmarking table comparing different material systems used for photodetection based on responsivity, detectivity, and noise equivalent power (PDF)
