# Supplementary material for: Encoding Light in Electronic Noise
Source: ACS Nano. 2026 Oct 5;20(41):27738–47. doi: 10.1021/acsnano.6c08996 (PMC13689905; doi:10.1021/acsnano.6c08996)
Supplement: Supplementary file 1 [file nn6c08996_si_001.pdf]

Encoding light in electronic noise

Arpan Ghosh[1], Seema Rani[1,2], Anshul Rasyotra[1], Samriddha Ray[1], Karthik G Krishnan[1], Md Sajjad Alam[3], Anirban Chowdhury[1], Kaushik Ghosh[2], and Saptarshi Das[1,3,4]*

[1]*Engineering Science and Mechanics, Penn State University, University Park, PA 16802, USA*

[2]*Institute of Nanoscience and Technology, Mohali,140306, India*

[3]*Electrical Engineering, Penn State University, University Park, PA 16802, USA*

[4]*Materials Science and Engineering, Penn State University, University Park, PA 16802, USA*

[*] *Corresponding Author*

Supporting Information 1

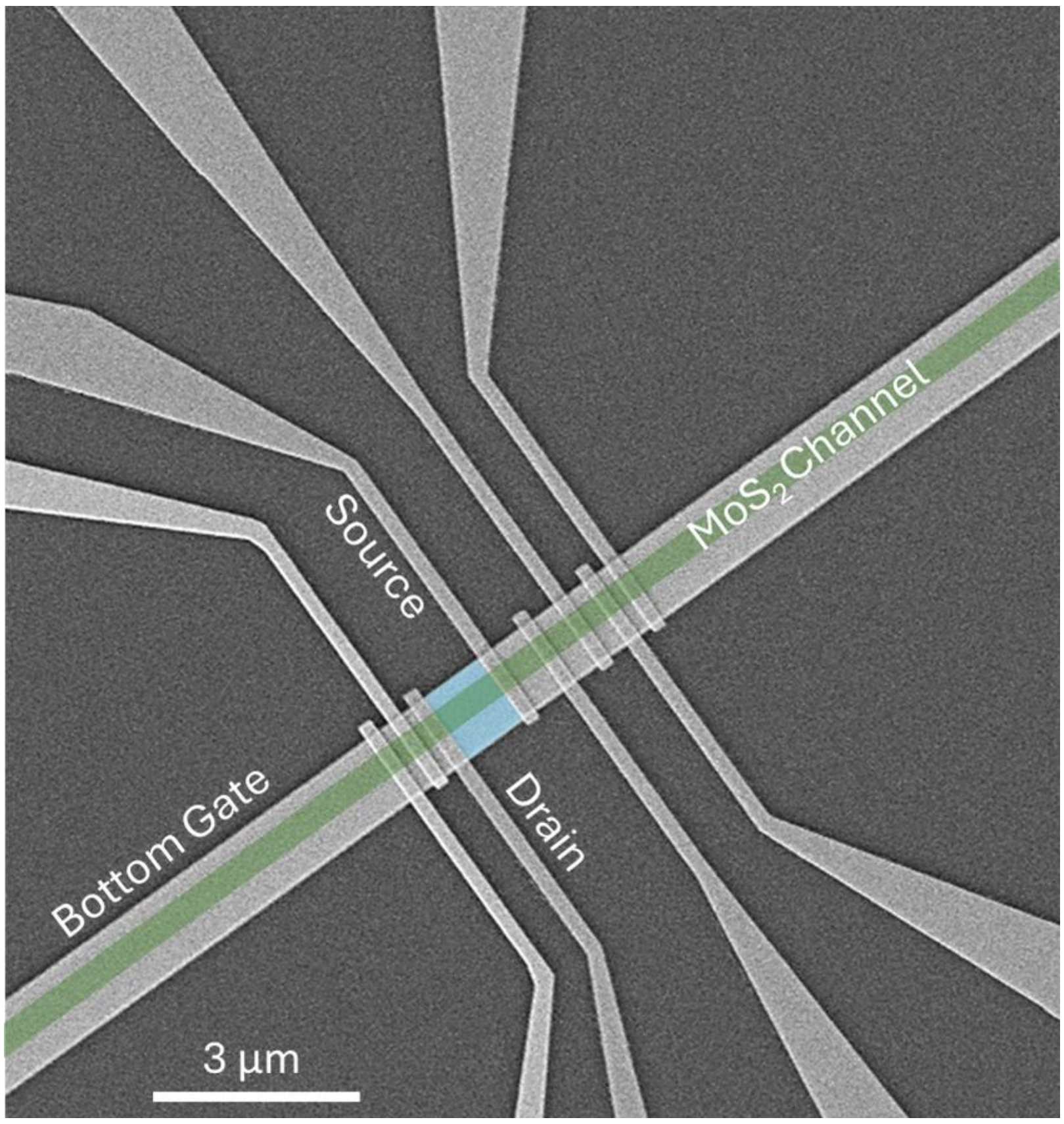

Supporting Information 1. Scanning electron microscopy (SEM) image of back-gated MoS_2 FET. *False-colored SEM image of a back-gated FET with monolayer MoS_2 as the semiconducting channel, and 5 nm HfO_2 as the back-gate dielectric.*

Supporting Information 2

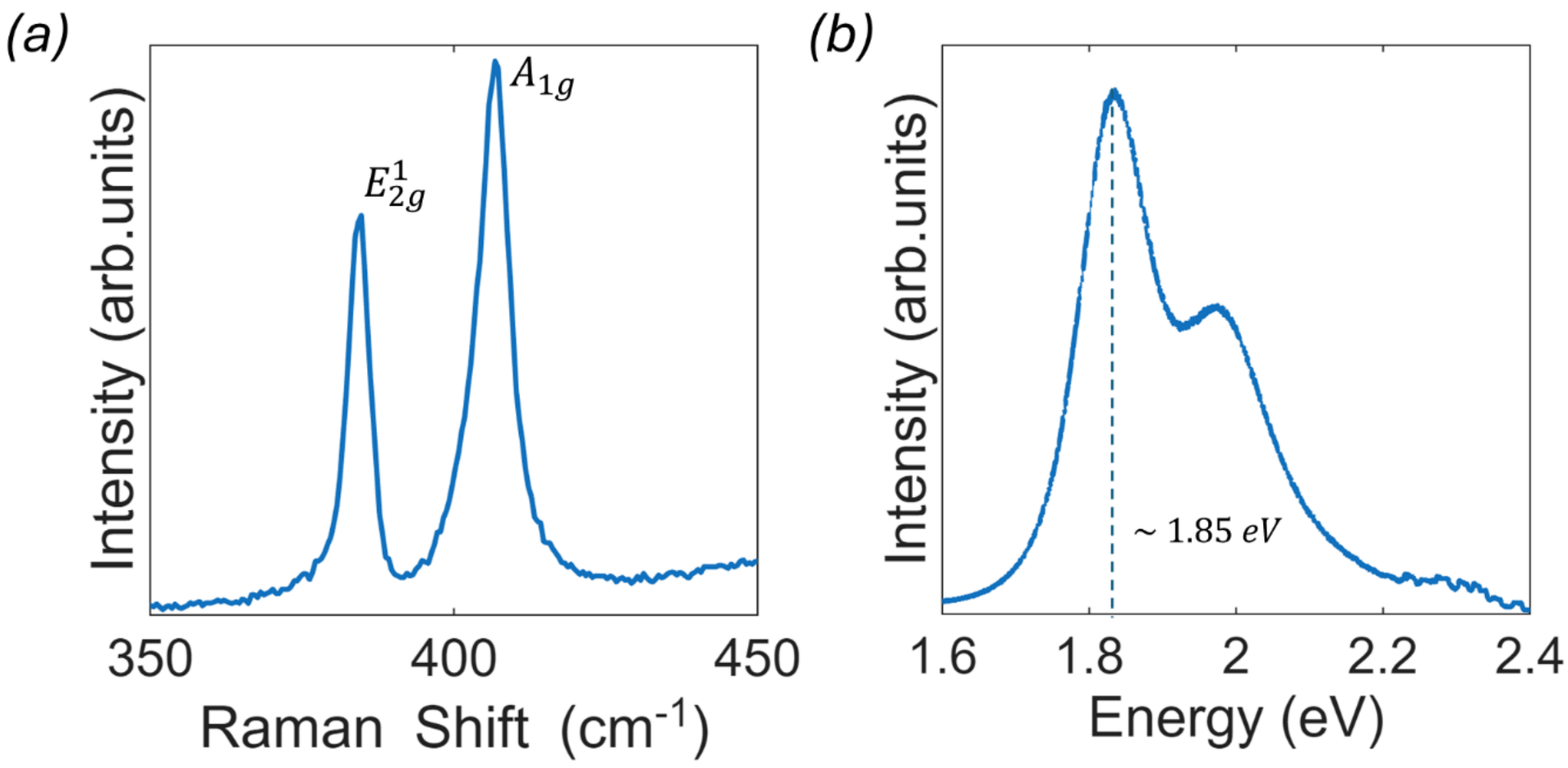

Supporting Information 2. MoS_2 characterization. (a) *Raman and* ***(b)*** *photoluminescence (PL) spectra for as-grown monolayer MoS_2 obtained using a 532 nm laser. The in-plan E^1_{2g} peak (384 cm^{-1}) out-of-plane of plane A_{1g} peak (407 cm^{-1}) Raman active vibration modes exhibit a peak difference of 17 cm^{-1}, confirming the monolayer nature of MoS_2 film. PL peak of the monolayer MoS_2 is observed at 1.85 eV, confirming the direct band gap from monolayer MoS_2 film.*

Supporting Information 3

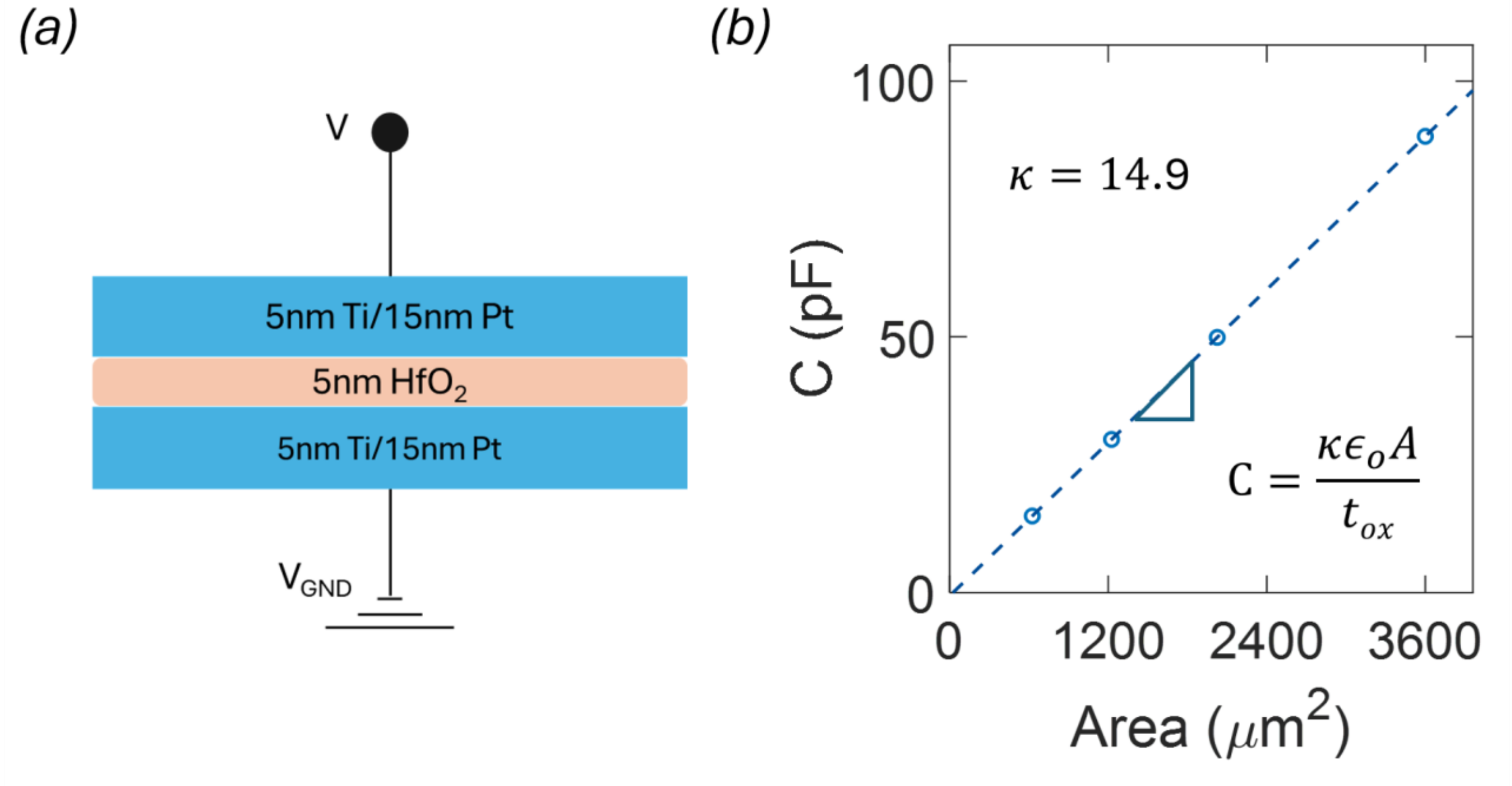

Supplementary Figure 3. Metal–insulator–metal (MIM) structure and dielectric characterization. (a) *Device schematic and **(b)** measured capacitance (C) versus capacitor area for MIM structures fabricated using 5 nm HfO_2. The linear scaling of capacitance with area enables extraction of the relative dielectric constant ($\kappa \approx 14.9$) from the slope of the curve. The corresponding equivalent oxide thickness (EOT) is given by $EOT = t_{ox}\frac{\kappa_{SiO_2}}{\kappa}$,yielding EOT*

Supporting Information 4

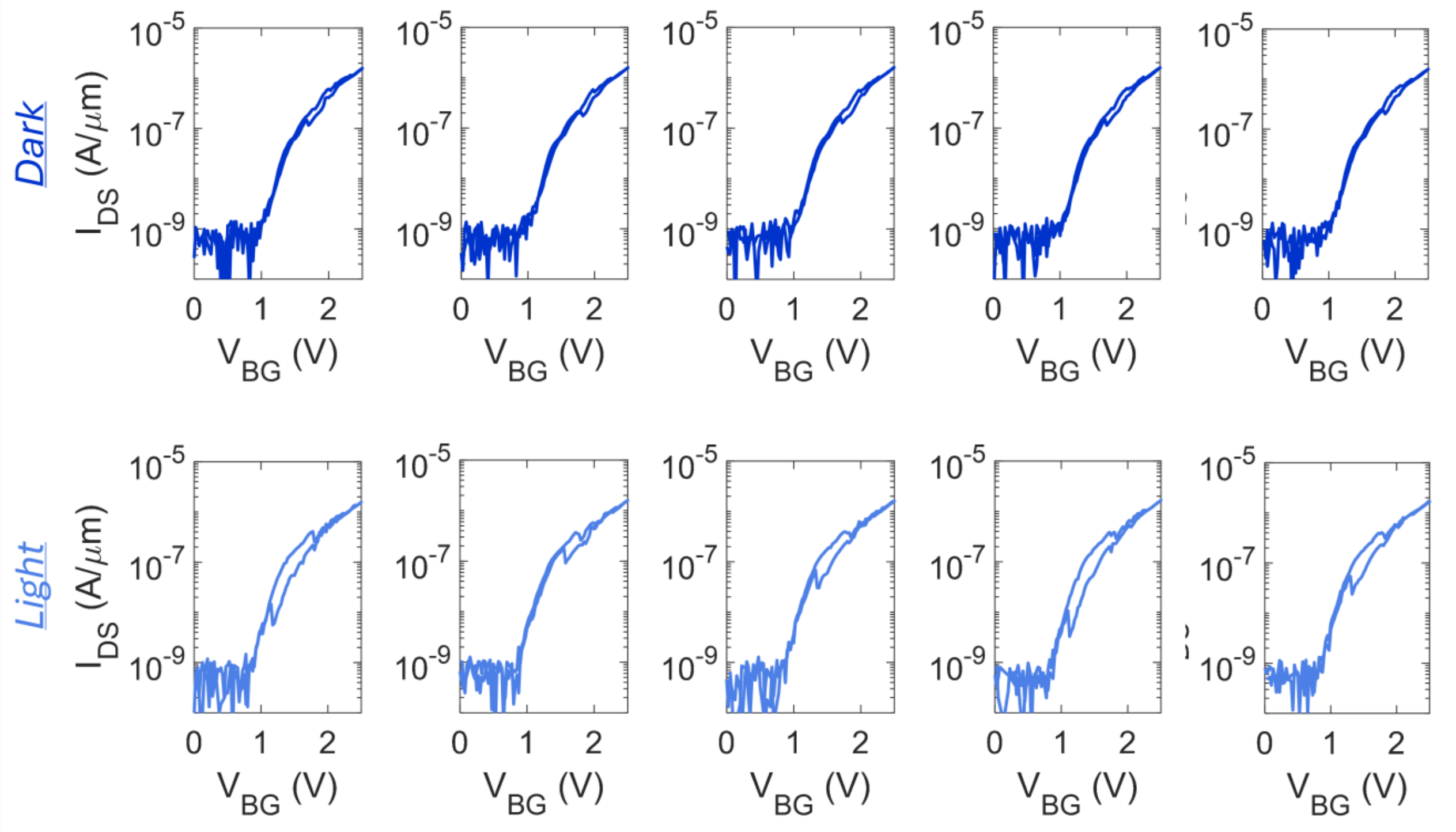

Supplementary Figure 4. Transfer characteristics of MoS_2 FET under dark and illumination at 15 K. *Back-gate transfer characteristics (I_{DS}–V_{BG}) measured over multiple consecutive sweeps from the same device at 15 K in the dark (top row) and under illumination (bottom row), at a fixed drain bias (V_{DS}*

Supporting Information 5

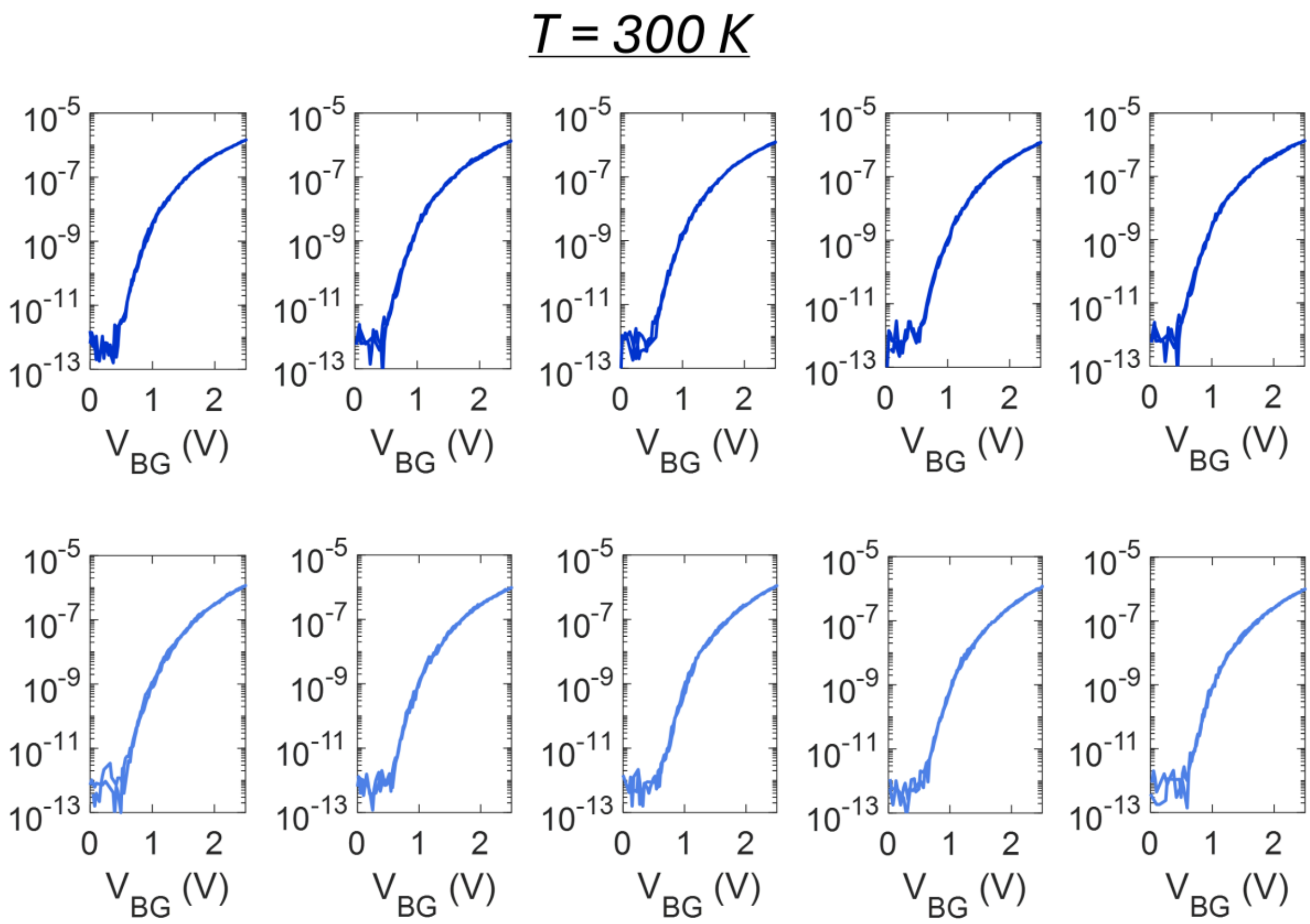

Supplementary Figure 5. Room-temperature transfer characteristics of MoS_2 FET under dark and illuminated conditions. *Back-gate transfer characteristics (I_{DS}–V_{BG}) measured over multiple consecutive sweeps from the same device at room temperature in the dark (top row) and under illumination (bottom row), at a fixed drain bias (V_{DS}*

Supporting Information 6

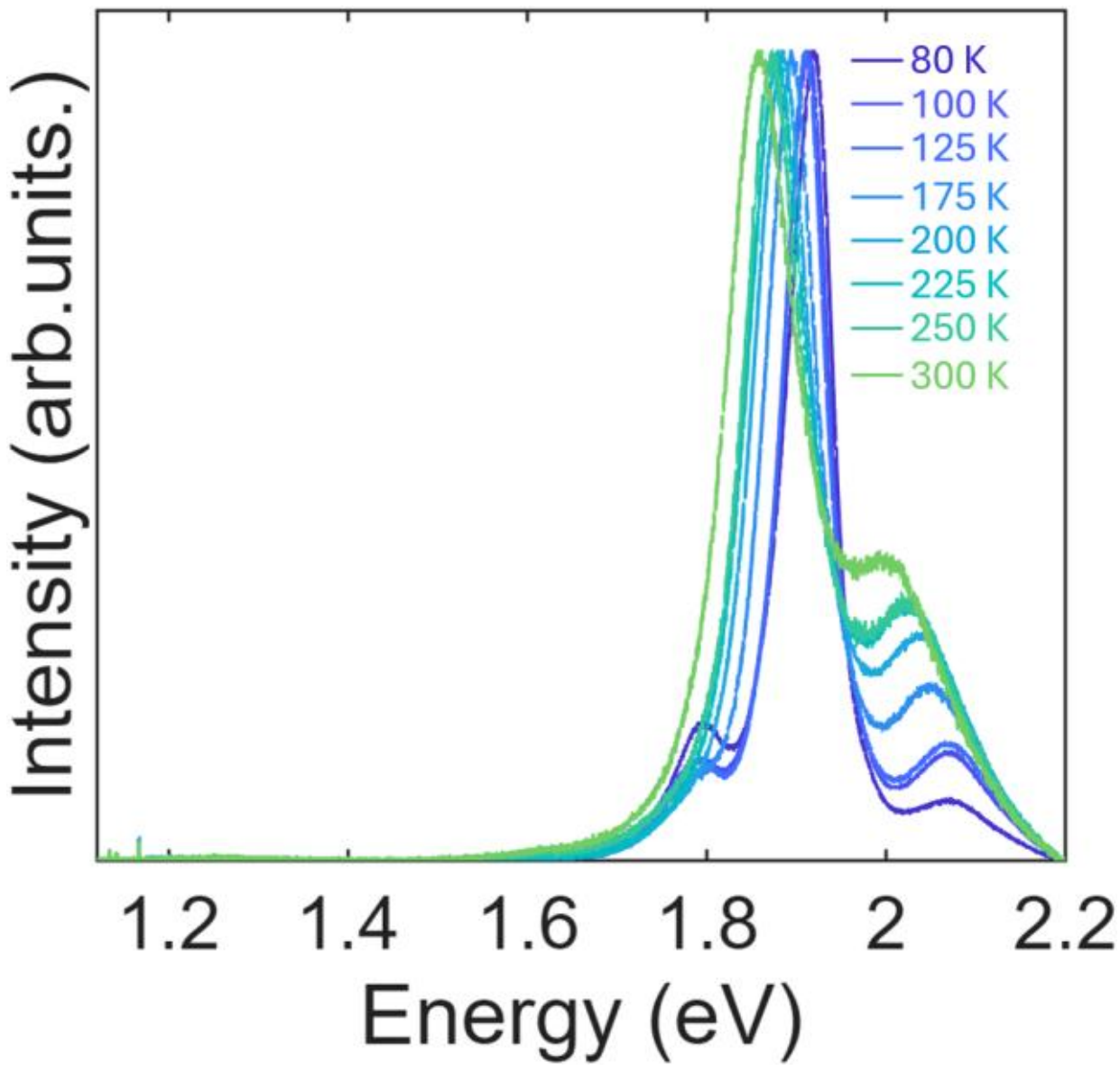

Supporting Information 6. Temperature-dependent photoluminescence (PL) of the MoS_2/HfO_2 interface. *Photoluminescence spectra of MoS_2 measured from 80 to 300K. A pronounced emission feature near 1.8 eV emerges at low temperatures and is progressively quenched with increasing temperature, becoming indistinguishable at 300K. This thermally quenched low-energy emission is consistent with defect- or trap-associated radiative recombination at the MoS_2/HfO_2 interface. In contrast, the higher-energy peak near 2.05 eV becomes increasingly prominent as the temperature rises. The temperature-dependent evolution of these spectral features, together with the observed peak shift, supports the presence of interfacial defect states in the MoS_2/HfO_2 heterostructure.*

Supporting Information 7

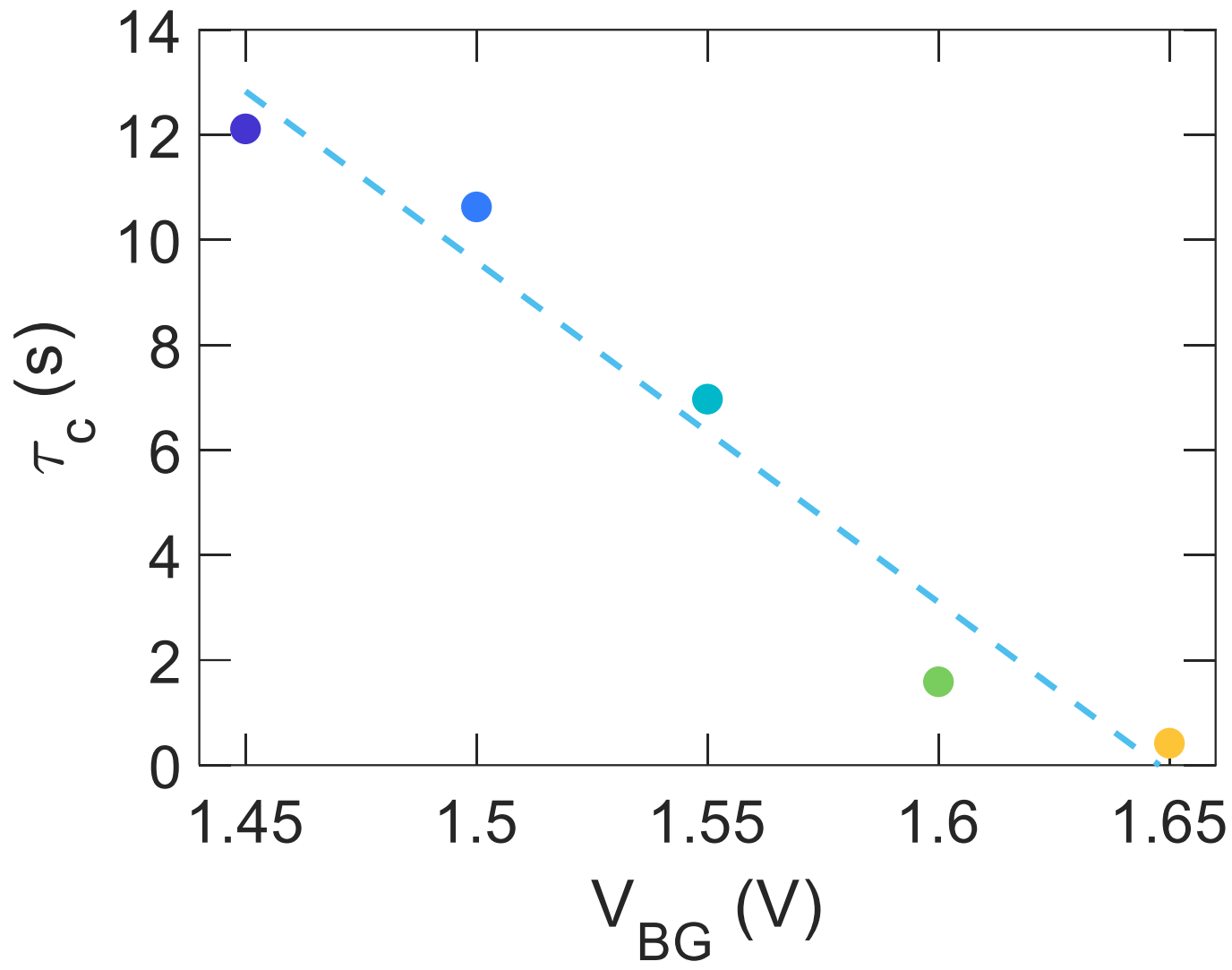

Supporting Information 7. Dependence of time to capture (τ_c) on back-gate bias (V_{BG}). *τ_c as a function of V_{BG} is extracted from time-domain measurements. τ_c decreases with increasing V_{BG}, indicating faster carrier capture into interfacial trap states.*

Supporting Information 8

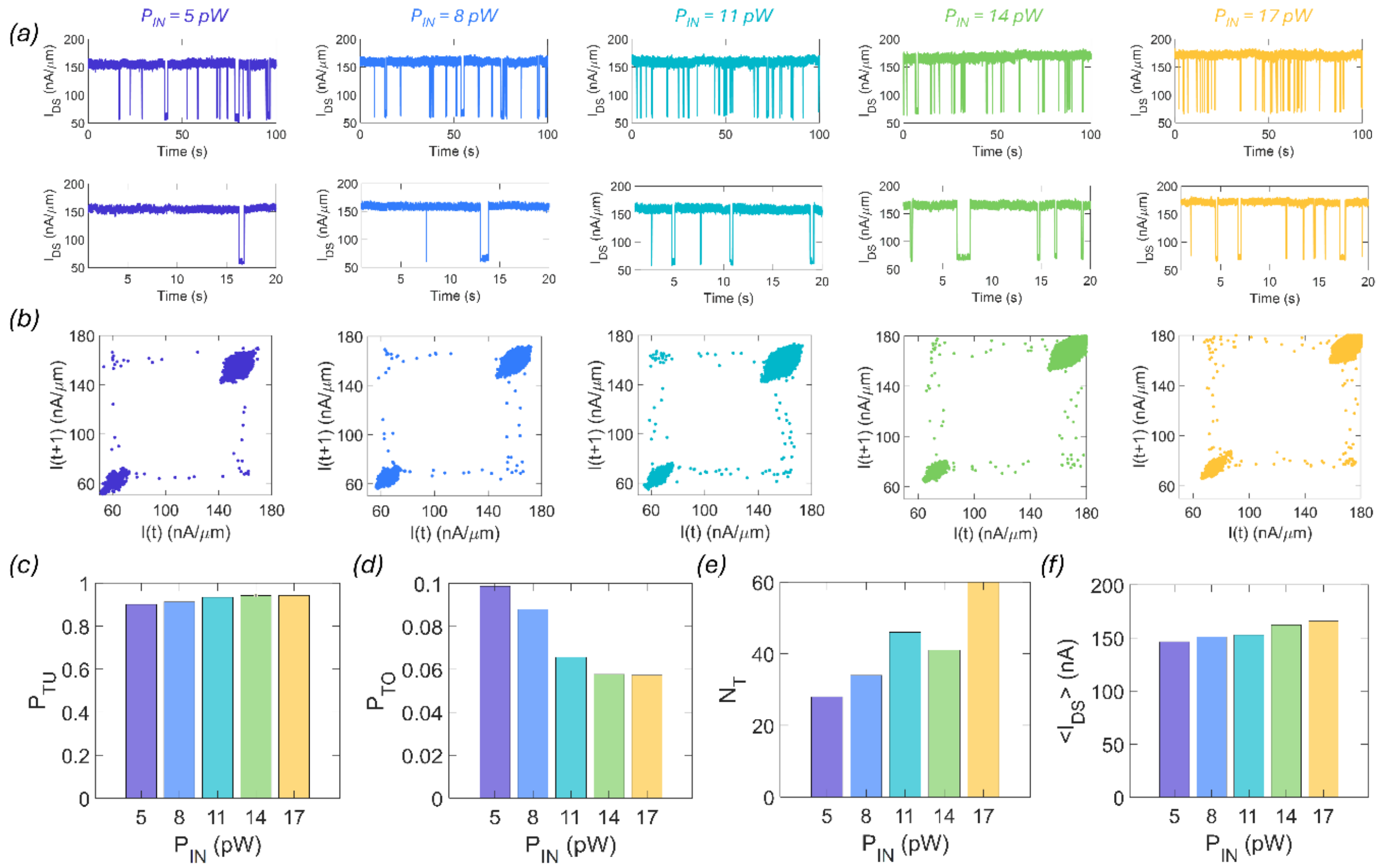

Supporting Information 8. Intensity-dependence of stochastic photo sensing. (a) *Time-resolved I_{DS} traces recorded under progressively increasing illumination power (P_{IN}) at a fixed V_{BG} = 1.5 V. Both longer dwell traces (top row) and shorter dwell windows (lower row) are shown to highlight the evolution of the switching dynamics.* ***(b)*** *Time-lag plots (TLPs) i.e. $I(t + \Delta t)$ versus $I(t)$ corresponding to the long-duration traces in* ***(a)****, shows clear bimodal clustering, a characteristic of two-state fluctuations. At lower P_{IN} ,the off-diagonal clusters are sparsely populated, indicating fewer state transitions. With increasing P_{IN}, off-diagonal population becomes denser, confirming a higher transition frequency. The occupancy probabilities for* ***(c)*** *trap-occupied (p_{TO}) and* ***(d)*** *trap-unoccupied (p_{TU}) states for different P_{IN}.* ***(e)*** *Total number of transitions, N_T versus P_{IN}, shows a monotonic increase consistent with an increased frequency of combined carrier capture and emission events under stronger optical excitation.* ***(f)*** *Expected drain current, $< I_{DS} >$ as a function of P_{IN}. The rise in $< I_{DS} >$ originates from the longer dwell time of the carrier in trap-unoccupied (high-current) state at higher illumination intensities.*

Supporting Information 9

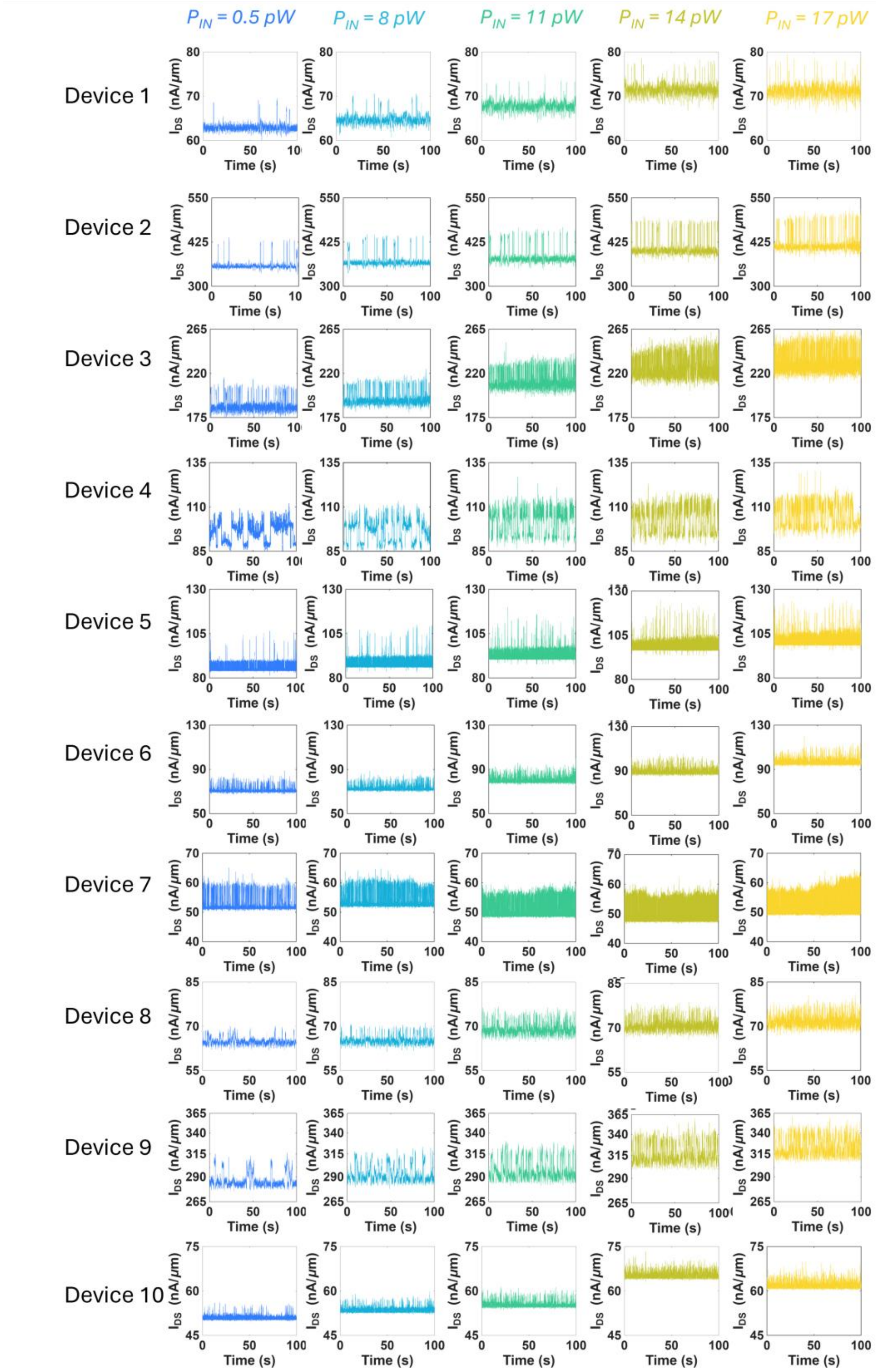

Supporting Information 9. Multiple MoS_2 FETs showcasing the stochastic light-induced RTNs. *Ten devices exhibit light-activated random telegraph noise (RTN), and the emission rate consistently increases with increasing illumination intensity, demonstrating that the key stochastic photosensing. behavior is reproducible and not limited to a single representative device.*

Supporting Information 10

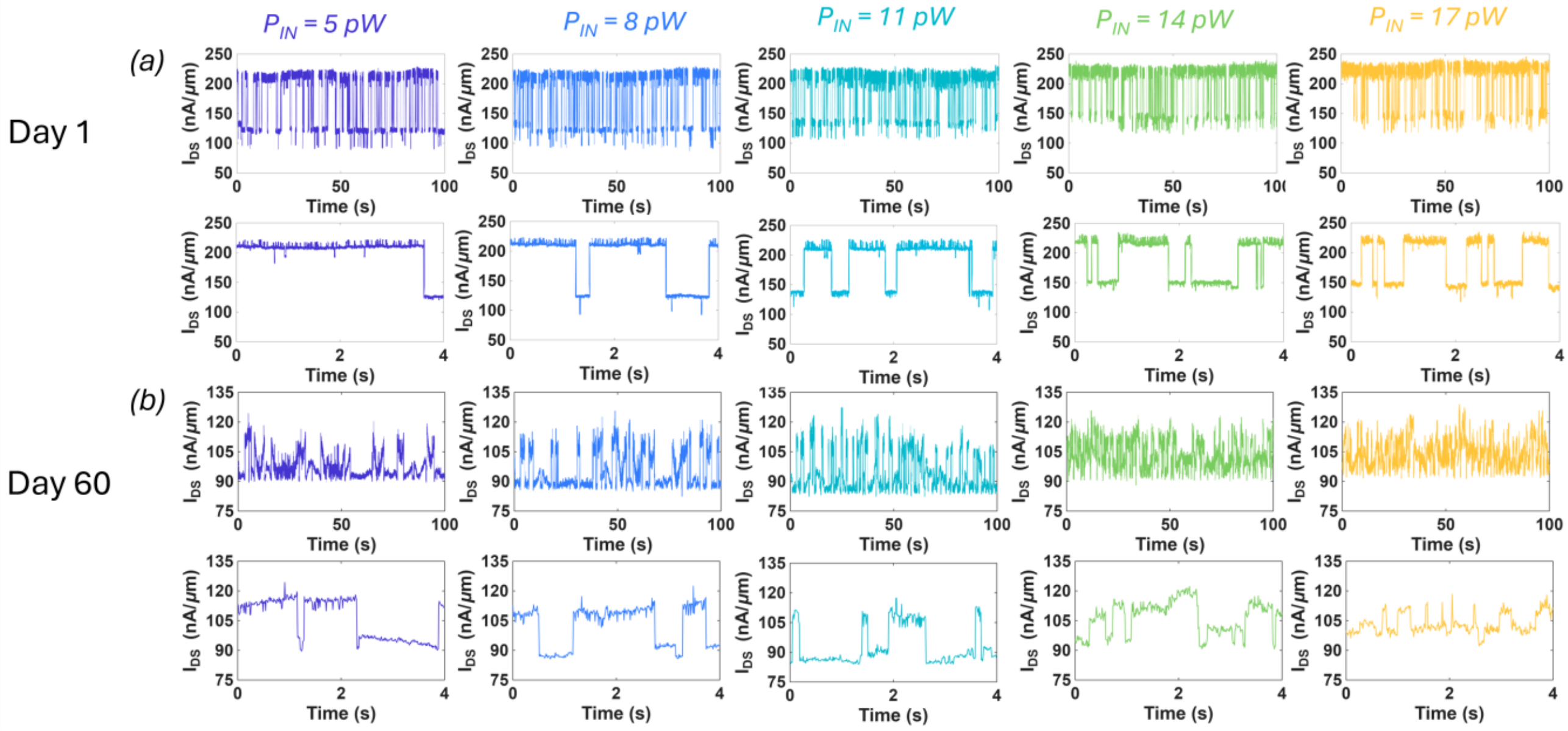

Supporting Information 10. Temporal stability of light-induced random telegraph noise (RTN). *Time-resolved drain-current (I_{DS}) traces measured on* ***(a)*** *Day 1 and* ***(b)*** *Day 60 under progressively increasing incident illumination power (P_{in}). The upper traces in each panel show the RTN response over 100 s, while the corresponding lower traces present 4s magnified views, highlighting the stochastic transitions and variations in dwell time between the discrete current states. The persistence of light-induced RTN after 60 days, together with its systematic evolution with increasing illumination power, demonstrates the long-term temporal stability and reproducibility of the phenomenon.*

Supporting Information 11

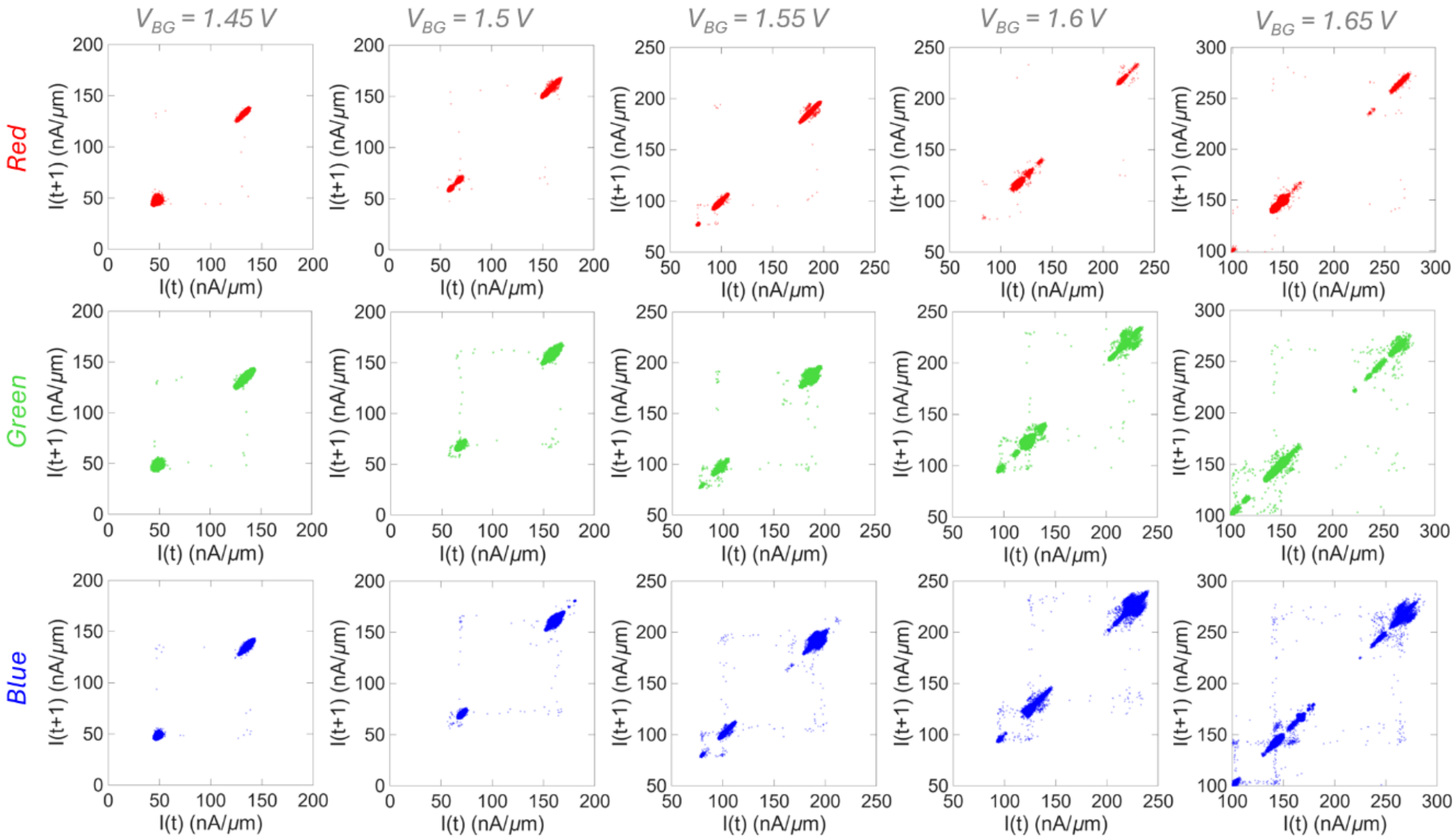

Supporting Information 11. Time-lag plots for visualizing the wavelength and gate bias dependence of stochastic dynamics in MoS_2 FET. *Time-lag plots (TLPs) i.e.* $I(t + \Delta t)$ *versus* $I(t)$ *corresponding to Figure 4 for red, green, and blue illumination at different gate biases (V_{BG}). For a fixed V_{BG}, the density of the off-diagonal elements increases with increasing photon energy, indicating an increase in the number of transitions. However, with increasing V_{BG}, emission is suppressed, which results in the system progressively spending more time in the lower-current state.*

Supporting Information 12

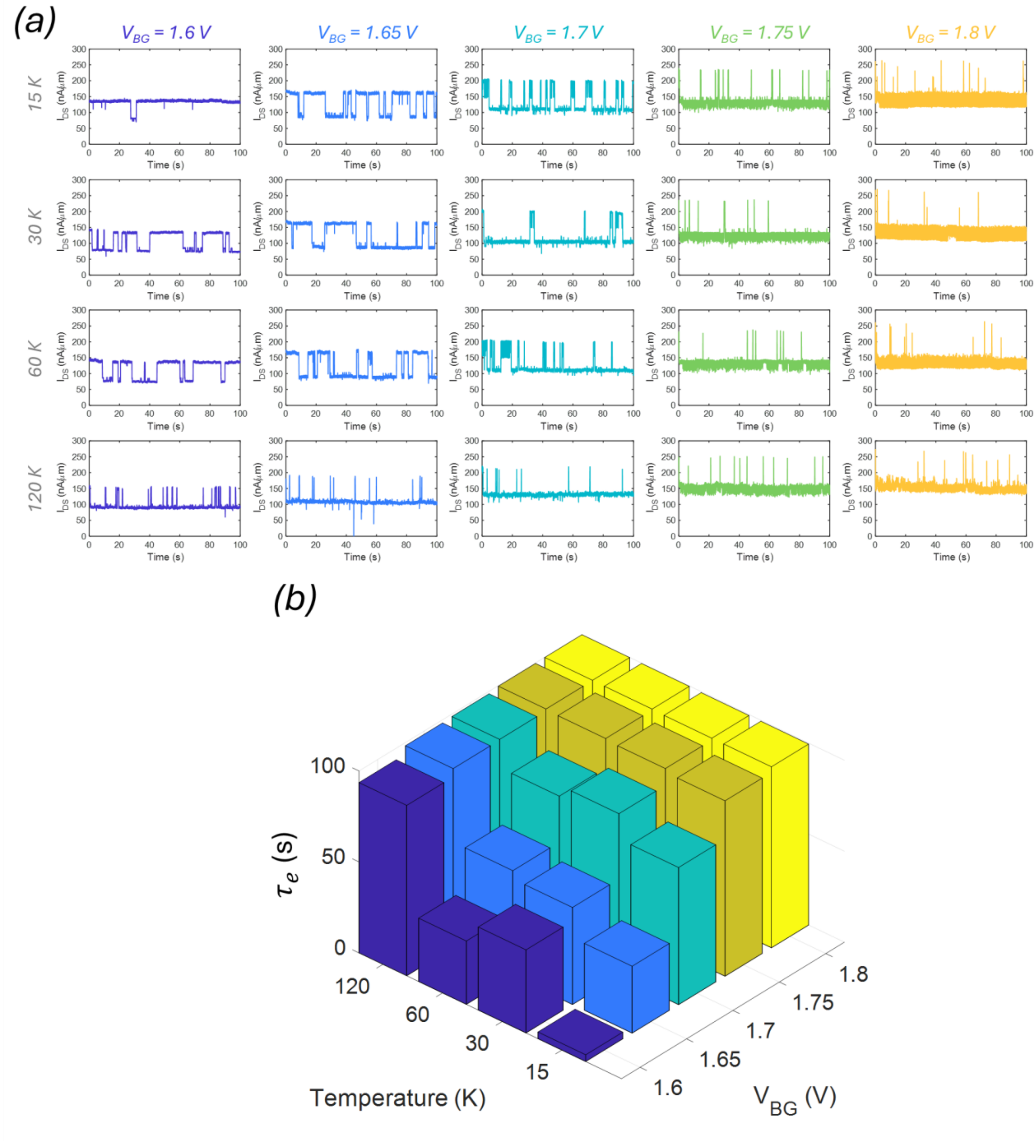

Supporting Information 12. Temperature dependence of stochastic switching dynamics. (a) *Time-resolved I_{DS} traces and **(b)** dwell time in capture state (τ_e) as a function of back-gate bias (V_{BG}) and temperature. A pronounced two-level switching is observed at lower temperature (15 K), which becomes progressively intermittent and suppressed with increasing temperature.*

Supporting Information 13

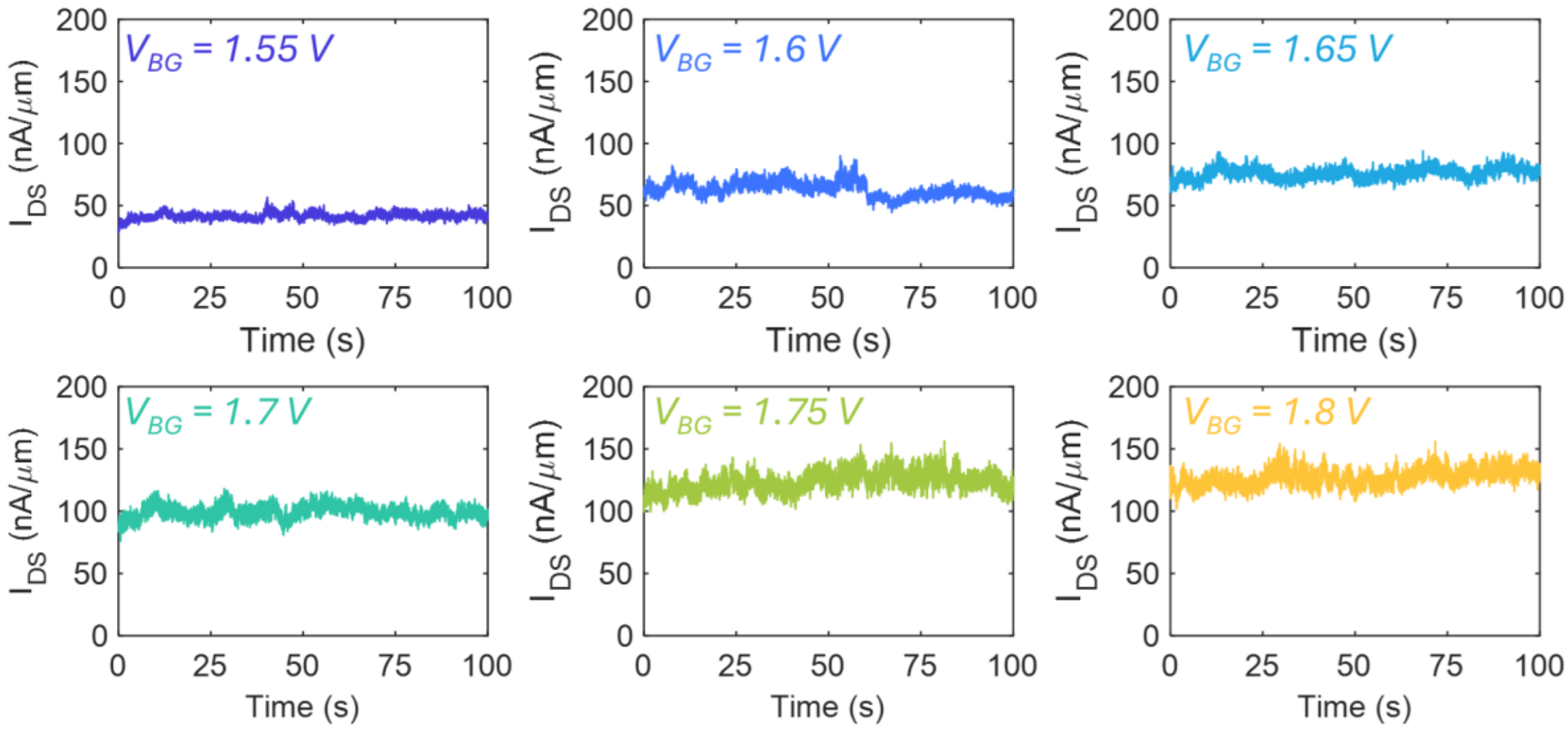

Supporting Information 13. Stochastic switching dynamics at room temperature. *At 300 K, the current remaining stable across all gate-biases (V_{BG}) with no RTN observed under illumination, indicating suppression of stochastic dynamics due to dominant thermal contribution.*

Supporting Information Table 1

Ref	Material System	Photo detection mechanism	λ (nm)	NEP $(W/\sqrt{Hz})$	R (AW^{-1})	D* (Jones)
[1]	β-In_2S_3	Photoconductive	450-900	-	137	4.74 x 10^{10}
[2]	ReS_2	Photoconductive	532	-	8.8 x 10^4	1.2 x 10^{12}
[3]	MoS_2	Photogating	400-680	1.8 x 10^{-15}	880	
[4]	WSe_2	Photogating	500-900	-	3.5 x 10^5	1 x 14^{14}
[5]	In_2Se_3	Photogating	405-940	-	9.8 x 10^4	3.3 x 10^{13}
[6]	PbS	-	800	-	1621	1.72 x 10^{11}
[7]	Graphene/WS_2	-	690	-	8.96 x 10^4	8.86 x 10^{11}
[8]	$MoTe_2$	Photoconductive	1550	90 x 10^{12}	0.5	
[9]	Black Phosphorous	-	3000-5000	4.35 x 10^{12}	-	4.9 x 10^9
[10]	MoS_2	Photogating	450-625	-	3.6 x 10^7	5.6 x 10^{13}
**This Work**	**1L-MoS_2/ HfO_2**	**Stochastic Sensing utilizing RTN**	**450-750**	**5.4 x 10^{-17}**	**7.9 x 10^5**	**1.86 x 10^{12}**

References

[1] W. Huang *et al.*, "Controlled synthesis of ultrathin 2D β-In2S3 with broadband photoresponse by chemical vapor deposition," *Advanced Functional Materials,* vol. 27, no. 36, p. 1702448, 2017.

[2] E. Liu *et al.*, "High responsivity phototransistors based on few-layer ReS2 for weak signal detection," *Advanced Functional Materials,* vol. 26, no. 12, pp. 1938–1944, 2016.

[3] O. Lopez-Sanchez, D. Lembke, M. Kayci, A. Radenovic, and A. Kis, "Ultrasensitive photodetectors based on monolayer MoS2," *Nature nanotechnology,* vol. 8, no. 7, pp. 497–501, 2013.

[4] W. Zhang, M.-H. Chiu, C.-H. Chen, W. Chen, L.-J. Li, and A. T. S. Wee, "Role of metal contacts in high-performance phototransistors based on WSe2 monolayers," *ACS nano,* vol. 8, no. 8, pp. 8653–8661, 2014.

[5] J. O. Island, S. I. Blanter, M. Buscema, H. S. van der Zant, and A. Castellanos-Gomez, "Gate controlled photocurrent generation mechanisms in high-gain In2Se3 phototransistors," *Nano letters,* vol. 15, no. 12, pp. 7853–7858, 2015.

[6] Y. Wen *et al.*, "Epitaxial 2D PbS nanoplates arrays with highly efficient infrared response," *Advanced Materials,* vol. 28, no. 36, pp. 8051–8057, 2016.

[7] T. He *et al.*, "Enhanced responsivity of a graphene/Si-based heterostructure broadband photodetector by introducing a WS2 interfacial layer," *Journal of Materials Chemistry C,* vol. 9, no. 11, pp. 3846–3853, 2021.

[8] R. Maiti *et al.*, "Strain-engineered high-responsivity MoTe2 photodetector for silicon photonic integrated circuits," *Nature Photonics,* vol. 14, no. 9, pp. 578–584, 2020.

[9] M. Long *et al.*, "Room temperature high-detectivity mid-infrared photodetectors based on black arsenic phosphorus," *Science advances,* vol. 3, no. 6, p. e1700589, 2017.

[10] A. Dodda *et al.*, "Active pixel sensor matrix based on monolayer MoS2 phototransistor array," *Nature Materials,* vol. 21, no. 12, pp. 1379–1387, 2022.
